# Exploring the therapeutic potential of naturally occurring taxifolin, a dietary flavonoid: an updated comprehensive review

**DOI:** 10.3389/fphar.2026.1780792

**Published:** 2026-03-25

**Authors:** Gehad H. Mandour, Ahmed M. El-Dessouki, Kareem A. Attallah, Mahmoud A. Seliem, Ahmed R. Abdullah, Emad Gamil Khidr, Ahmed A. El-Husseiny, Riham A. El-Shiekh, Mohamed M. Hafez, Hazim O. Khalifa

**Affiliations:** 1 Department of Pharmacognosy, Faculty of Pharmacy, Ahram Canadian University, Giza, Egypt; 2 Pharmacology and Toxicology Department, Faculty of Pharmacy, Ahram Canadian University, Giza, Egypt; 3 Research and Development Department, Biotechnology Research Center, New Damietta, Egypt; 4 Clinical Research Department, Damietta Directorate for Health Affairs, Egyptian Ministry of Health and Population, Damietta, Egypt; 5 Department of Biochemistry, Faculty of Pharmacy, Ahram Canadian University, Giza, Egypt; 6 Biochemistry and Molecular Biology Department, Faculty of Pharmacy, Al-Azhar University, Cairo, Egypt; 7 Department of Biochemistry, Faculty of Pharmacy, Egyptian Russian University, Cairo, Egypt; 8 Department of Pharmacognosy, Faculty of Pharmacy, Cairo University, Cairo, Egypt; 9 Department of Veterinary Medicine, College of Agriculture and Veterinary Medicine, United Arab Emirates University, Al Ain, United Arab Emirates; 10 United Arab Emirates University (UAEU) Center for Public Policy and Leadership, United Arab Emirates University, Al Ain, United Arab Emirates

**Keywords:** anti-inflammatory, antioxidant, flavonoids, functional foods, Nutraceuticals, taxifolin

## Abstract

**Purpose:**

Flavonoids, well-known as key bioactive compounds in numerous medicinal plants, help protect these plants against both biotic and abiotic stresses and are linked to the prevention of various degenerative diseases. The diverse pharmacological effects and therapeutic potential of flavonoids are influenced by factors such as their level of hydroxylation, structural classification, additional substitutions and conjugations, extent of polymerization, and their ability to chelate metals.

**Methods:**

Data from various databases such as the Egyptian Knowledge Bank (EKB), Scopus, Web of Science, PubMed, Google Scholar, and Elsevier databases were gathered until April 2025. All possible keywords pertaining to taxifolin, natural origins, isolation, structure, solubility, synthesis, bioavailability, applications, biological activities, mechanisms of actions, pharmacokinetics, and clinical studies were utilized in the search.

**Results:**

Taxifolin, a bioactive flavonoid commonly found in dietary sources such as onions, milk thistle, and Douglas fir bark, has garnered significant attention for its extensive health-promoting properties. It exhibits potent antioxidant, anti-inflammatory, anticancer, antimicrobial, cardioprotective, neuroprotective, and hepatoprotective effects. Notably, taxifolin demonstrates superior antioxidant capacity linked to its phenolic hydroxyl groups and structural features, enabling effective free radical scavenging.

**Conclusion:**

Despite these promising pharmacological activities, further research is necessary to elucidate its detailed molecular mechanisms, pharmacokinetic profile, and comprehensive safety through well-designed randomized clinical trials to facilitate its development as a therapeutic agent for human use.

## 1 Introduction

Flavonoids are commonly found secondary metabolites characterized by low molecular weight hydroxylated phenolic compounds. Typically, they exist in plants as aglycones, glycosides, or methylated derivatives, contributing various color hues such as blue, scarlet, and orange to leaves, flowers, and fruits (Jain and Vaidya, 2023). Taxifolin (3,5,7,3,4-pentahydroxy flavanone), a naturally occurring flavonoid also known as dihydroquercetin, is a compound found in a variety of plant species such as milk thistle (Wallace et al., 2005), onions (Slimestad et al., 2007), Douglas fir bark (Kiehlmann and Li, 1995), and French maritime pine bark (Rohdewald, 2002). It was first isolated from Douglas fir bark (*Pseudotsuga taxifolia* (Lindl.) Britton) and later Dahurian and Siberian larch (*Larix sibirica* Ledeb. and *Larix gmelinii* (Rupr.) Kuzen.) (Sunil and Xu, 2019). Taxifolin has similar pharmacological effects to other flavonoids, including anti-inflammatory, antioxidant, cardioprotective, and anticancer properties. The most fundamental role of taxifolin among which are antioxidant and anti-inflammatory properties (Jain and Vaidya, 2023; Sunil and Xu, 2019; Das et al., 2021; Liu et al., 2023a). This article reviews the pharmacological activities of taxifolin and highlights recent advancements in its applications for treating several chronic diseases, while also exploring its potential therapeutic uses moving forward.

### 1.1 Search strategy

Data from various databases such as the Egyptian Knowledge Bank (EKB), Scopus, Web of Science, PubMed, Google Scholar, and Elsevier databases were gathered until April 2025. All possible keywords pertaining to taxifolin; natural origins, isolation, structure, solubility, synthesis, bioavailability, applications, biological activities, mechanisms of actions, pharmacokinetics, and clinical studies were utilized in the search. There was no defined time frame for data collection; all relevant data were gathered comprehensively.

## 2 Phytochemistry of taxifolin

### 2.1 Natural sources

Taxifolin is a secondary metabolite that is broadly distributed throughout the plant kingdom. It is found in particularly high concentrations within plants from the Pinaceae family where Siberian larch (*L. sibirica*) (Lee et al., 2007), Douglas fir (*Pseudotsuga menziesii*) (Kiehlmann and Li, 1995), and Himalayan cedar (*Cedrus deodara*) (Kumar et al., 2021) are among the notable members. Taxifolin exists naturally in milk thistle (*Silybum marianum*) (Wallace et al., 2005), onions (*Allium cepa*) (Slimestad et al., 2007), olive oil (*Olea europaea*) (De Marino et al., 2014), and *Stizolophus balsamita* (costmary or alecost) (Nawrot et al., 2021). Additionally, taxifolin can be derived from silymarin, a complex extract obtained from milk thistle seeds (Kim et al., 2024) and has also been detected in vinegar that has been aged in cherry wood (Cerezo et al., 2014).

### 2.2 Chemical structure

**FIGURE 1:**
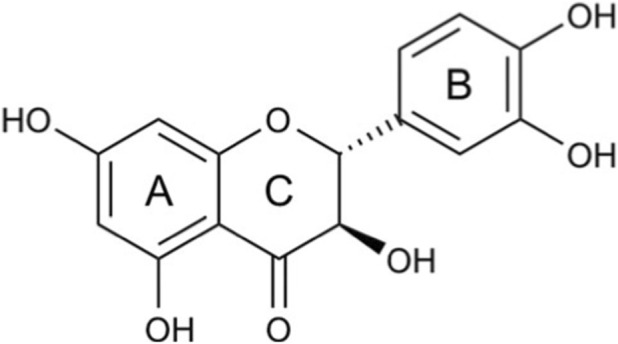
Chemical structure of taxifolin.

The chemical structure of taxifolin is defined by its molecular formula (C_15_H_12_O_7_), molecular weight of 304.25 g/mol (Jain and Vaidya, 2023; Nifant’ev et al., 2006), systematic IUPAC name is (2R,3R)-2-(3,4-dihydroxyphenyl)-3,5,7-trihydroxy-2,3-dihydrochromen-4-one (Weidmann, 2012), known as (2R,3R)-3,3′,4′,5,7-Pentahydroxyflavan-4-one (Vinayagamurthy et al., 2024), and more commonly as dihydroquercetin (Wallace et al., 2005) (Figure 1). Taxifolin belongs to the flavanonol subclass of flavonoids, which are part of the broader group of polyphenolic compounds. The C15 framework consists of two benzene rings (A and B) joined by a heterocyclic ring (C) as its fundamental structure. The antioxidant properties of taxifolin are supported by five hydroxyl groups placed at positions 3, 5, 7, 3' and 4' that enable effective free radical scavenging. Taxifolin contains two stereocenters at positions 2 and 3 of its C-ring which produces multiple stereoisomeric forms (Jain and Vaidya, 2023).

### 2.3 Isolation and extraction methodologies

Multiple documented procedures exist for extracting taxifolin from its natural origin. The extraction of taxifolin typically requires organic solvents with ethanol as the primary choice. Taxifolin extraction from larch roots requires 90% ethanol solution under heating and reflux conditions. The extract undergoes concentration followed by hot water solution before the solution crystallizes through cooling. The extraction method can be executed repeatedly to boost the purity levels (Liu et al., 2021). The extraction process of taxifolin from plant materials including *Abies nephrolepis* leaves and bark has been improved using ultrasound-assisted extraction methods. Ultrasonic waves disrupt plant cell walls through ultrasonic waves to enable the target compounds to release into the solvent (Wei et al., 2020). Other studies described taxifolin extraction from boiled water extracts of *Trichilia emetica* whole seeds using flash chromatography. The separation technique uses a C18 column as the stationary phase while a mobile phase consisting of a linear methanol and water mixture containing 0.1% formic acid to determine compound affinities. The column separation process leads to the collection of fractions that contain taxifolin (Usman et al., 2016). The selection process for extraction methods and solvents depends on three main factors: the plant material type, the production scale requirements and the purity standards for the end product. Taxifolin can be obtained in different polymorphic forms through recrystallization procedures that utilize ethanol, methanol and ethyl acetate as different solvents.

### 2.4 Spectroscopic data

Spectroscopic methods serve as essential tools for identifying and characterizing taxifolin when isolated. Mass spectrometry (MS) generates essential data about compound molecular weight and its breakdown patterns (Table 1). The isolated taxifolin from *Larix olgensis* displayed a molecular ion peak at m/z 305.0673 [M + H]+ and its characteristic fragment ions were also observed (Zhou et al., 2018). The mass spectrum of taxifolin isolated from *T. emetica* matched its predicted molecular weight (Usman et al., 2016). The analysis through infrared (IR) spectroscopy shows which functional groups exist in the molecule. Taxifolin displays characteristic absorption peaks at 3,428 cm^−1^ for O-H stretching while C-H stretching occurs at 2,953 and 2,833 cm^−1^ and carbonyl (C=O) stretching appears at 1,636 cm^-1^ and the spectrum contains bands associated with aromatic C=C stretching. Nuclear Magnetic Resonance (NMR) spectroscopy uses ^1^H NMR and ^13^C NMR to analyze atomic nuclei magnetic properties for obtaining detailed structural information about molecules. The ^1^H NMR spectrum of taxifolin shows distinct signals representing all its protons which include aromatic ring and heterocyclic C-ring protons with unique chemical shift positions and coupling relationships. The ^13^C NMR spectrum shows all carbon environments that exist within the molecule structure. Scientists use these spectroscopic data to verify the identity and purity of isolated taxifolin by comparing them to reported values in scientific literature (Usman et al., 2016; Zhou et al., 2018). The combination of high-performance liquid chromatography with tandem mass spectrometry through HPLC-MS/MS provides a valuable method for identifying taxifolin and other flavonoids in complex mixtures (Zhou et al., 2018).

**TABLE 1:** Spectroscopic characterization data of taxifolin.

Spectroscopic method	Key peaks/Values	References
MS	*m/z* 305.0673 [M + H]^+^, 322.0939 [M + NH_3_+H]^+^, 327.0493 [M + Na]^+^; Fragments at *m/z* 287.0545, 259.0545, 195.0284, 153.0178	Zhou et al. (2018)
IR (KBr)	3,428 (O-H stretch), 2,953, 2,833 (C-H stretch), 1,636 (C=O stretch), 1,610, 1,510 (aromatic C=C stretch), 1,473, 1,415, 970, 775 cm^-1^	Zhou et al. (2018)
^1^H NMR (DMSO-d_6_)	δ 11.89 (s, 1H, OH-5), 10.84 (s, 1H, OH-7), 9.04 (s, 1H, OH-4'), 8.99 (s, 1H, OH-3'), 6.72 (d, 2H, H-5', 6'), 5.90 (d, 1H, *J* = 2 Hz, H-8), 5.85 (d, 1H, *J* = 2 Hz, H-6), 5.75 (d, 1H, *J* = 11 Hz, OH-3), 4.96 (d, 1H, *J* = 11 Hz, H-2), 4.48 (dd, 1H, *J* = 11, 6.0 Hz, H-3)	Zhou et al. (2018)
^13^C NMR (DMSO)	δ 197.68 (C4), 167.94 (C7), 163.30 (C5), 162.53 (C9), 145.75 (C3'), 144.92 (C4'), 128.02 (C1'), 119.36 (C6'), 115.33 (C5'), 115.09 (C2'), 100.40 (C10), 95.99 (C6), 95.00 (C8), 83.02 (C2), 71.54 (C3)	Zhou et al. (2018)

### 2.5 Physicochemical properties

Taxifolin shows low water solubility because its equilibrium solubility reaches 1.2 mg/mL (Stenger Moura et al., 2021). Taxifolin shows enhanced dissolving properties in polar organic solvents including ethanol and acetic acid and hot water (Chemical book, 2022). Its low solubility in water creates difficulties when formulating and delivering taxifolin in pharmaceutical and cosmetic products. Taxifolin shows thermal instability at elevated temperatures since its decomposition occurs during the melting process at 228 °C ± 1 °C. Taxifolin shows multiple crystalline forms, often described as polymorphism (Terekhov et al., 2020). Taxifolin exists in three distinct crystal states which are anhydrous without water molecules and hydrated with water molecules in the crystal lattice and amorphous as non-crystalline material. Different polymorphs of these compounds demonstrate distinct physicochemical properties which include solubility characteristics and stability levels and dissolution rate properties (Terekhov et al., 2020; Stenger Moura et al., 2021). During solubility studies taxifolin anhydrous transforms into its hydrate form (Stenger Moura et al., 2021). Taxifolin shows sensitivity to alkaline degradation, and its thermal breakdown occurs more rapidly when exposed to humidity according to stability tests. The compound demonstrates photostability when exposed to light and especially ultraviolet (UV) radiation (Moura et al., 2021). The stability characteristics of taxifolin need consideration for developing suitable storage methods and formulation techniques. The low water solubility and instability of taxifolin have led researchers to develop different formulation methods using zein-caseinate nanoparticles and selenized liposomes as nanocarriers. The developed systems demonstrate potential for enhancing taxifolin’s solubility properties while maintaining stability which leads to better bioavailability (Li et al., 2023; Qi et al., 2025).

### 2.6 Biosynthesis pathway in plants

**FIGURE 2:**
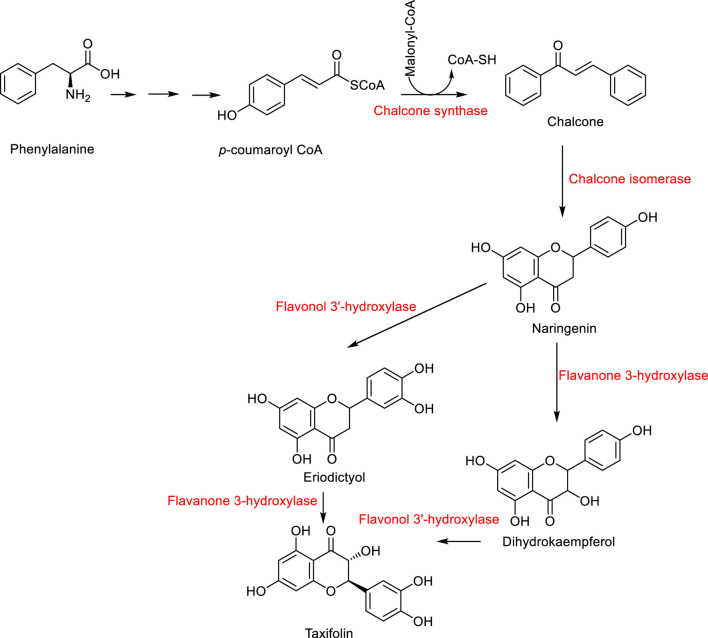
Biosynthesis of taxifolin.

Plants use taxifolin biosynthesis as a fundamental process within their extensive flavonoid biosynthesis network that starts from the phenylpropanoid metabolic pathway (Figure 2). The phenylalanine amino acid initiates the biosynthetic sequence that produces chalcones which serve as the building blocks for all flavonoids. After isomerization of chalcone produces a flavanone molecule it gets hydroxylated through the action of flavanone 3-hydroxylase (F3H) to form a dihydroflavonol (Liu et al., 2021). Taxifolin biosynthesis in Norway spruce (*Picea abies*) involves the F3H enzyme (*Pa*F3H) which converts the flavanone eriodictyol into the dihydroflavonol taxifolin. The flavonoid biosynthesis pathway includes dihydroflavonols as primary junction points since these compounds can evolve into different flavonoid groups including flavonols and anthocyanidins and flavan-3-ols. The defense compound catechin originates from taxifolin in Norway spruce (Taxifolin functions as a metabolic precursor for catechin). The key enzyme F3H shows altered expression levels during flavonoid biosynthesis pathway because environmental stresses and pathogen attacks affect its operation. This indicates taxifolin production functions as part of the plant defense system (Hammerbacher et al., 2019).

### 2.7 Quantification methods

Scientific methods exist to measure taxifolin concentrations in plant extracts as well as other sample matrices. HPLC operates as a common analytical method which works together with UV detection, photodiode array (PDA) detection, and MS. The analysis of phenolic compounds including taxifolin commonly uses HPLC with UV detection (HPLC-UV) because of its basic operational nature and widespread instrument availability. The extract separation process in this method depends on how components interact with stationary and mobile phases before UV light-based detection takes place (Almeida et al., 2016). For example, the analysis of taxifolin in larch root extract utilized HPLC with a UV detector was set at 288 nm at one study (Liu et al., 2021). Also, an established and verified an HPLC-PDA method for taxifolin quantification in *Pinus pinaster* bark extract was set at the same wavelength and proved beneficial for quality control laboratory work (Almeida et al., 2016).

For more complex samples or when higher sensitivity and selectivity are required, HPLC coupled with mass spectrometry (HPLC-MS) or tandem mass spectrometry (HPLC-MS/MS) is often utilized (Liu et al., 2021). MS detection allows for the identification and quantification of taxifolin based on its mass-to-charge ratio, providing greater specificity compared to UV detection (Almeida et al., 2016). Taxifolin extracted from *Larix olgensis* was quantified using HPLC-UV/ESI-MS, where the mass spectrometer confirmed the compound’s identity (Liu et al., 2021).

Reverse-phase HPLC (RP-HPLC) is a common mode of HPLC used for separating and quantifying taxifolin (Pozharitskaya et al., 2009). Methods have been developed for the simultaneous quantification of taxifolin and its glycosides, such as taxifolin 3-*O*-rhamnoside, using RP-HPLC with specific mobile phase compositions and detection wavelengths. A recent study developed and validated an RP-HPLC technique for the simultaneous measurement of taxifolin and taxifolin 3-*O*-rhamnoside in *Smilax china* Linn. rhizomes, demonstrating improved specificity, precision, and accuracy (Subramanian and Ramachandran, 2022).

The validation and development of these quantification methods serve to guarantee both plant extract quality control and product quality control that contains taxifolin and enables pharmacokinetic research. The reliability of a method depends on validation parameters which include linearity tests together with accuracy and precision measurements and detection and quantification limits.

## 3 Pharmacological activities of taxifolin (pre-clinical data)

Taxifolin’s intricate structure confers a range of pharmacological effects (Sarg et al., 2024). Several studies have highlighted its capacity to mitigate inflammation and oxidative stress, two major mechanisms linked to several chronic ailments, such as metabolic, neurological, and cardiovascular disorders (Liu et al., 2021; Obeidat et al., 2022). Accordingly, the following sections describe how its core pathways manifest in different pathological contexts rather than representing unrelated pharmacological actions.

### 3.1 Anti-cancer and immunomodulatory activities

The consumption of flavonoid-rich dietary products lowers the risk of cancer. Although the cancer-protective mechanisms of flavonoids are yet unknown, researchers believe that their fatty acid synthase (FAS) inhibitory characteristics may be responsible for inducing apoptosis in cancer cells (Brusselmans et al., 2005).

**FIGURE 3:**
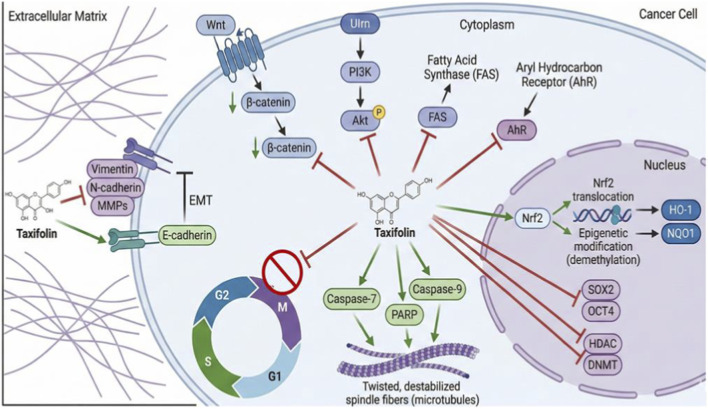
Mechanism of anticancer activity of taxifolin.

Taxifolin has been described as a powerful chemotherapeutic drug with strong antiproliferative actions against many cancer cells (Figure 3). Taxifolin inhibits fatty acid synthase in cancer cells, which restricts their proliferation and spread (Haque et al., 2018). Taxifolin increased the expression of phase II detoxifying and antioxidant enzymes through Nrf2 signaling. Notably, taxifolin promotes nuclear HO-1 expression in the cytoplasm and nuclear translocation by increasing Nrf2 expression. Taxifolin also regulates genes, inhibits mitosis in ovarian cancer cells (Kuang et al., 2017), and induces apoptosis in prostate cancer cells (Luo et al., 2008).

Kuang et al. (2017), a Chinese researcher, studied the effects of taxifolin on Nrf2 in JB6 P+ cells (a cutaneous keratinocyte cell line). The results showed that Nrf2 and its downstream genes, HO-1 and NAD(P)H quinone oxidoreductase 1 (NQO1), had increased messenger ribonucleic acid (mRNA) and protein levels. Taxifolin also reduced 12-O-tetradecanoylphorbol-13-acetate (TPA)-induced colony formation and antioxidant response element-luciferase activity. In addition, taxifolin suppressed the expression of histone deacetylase (HDAC) and DNA methyltransferase (DNMT) proteins in the western blotting investigation. The study’s findings significantly supported the use of the taxifolin-induced Nrf2 driven epigenetic pathway to treat skin cancer. Furthermore, Taxifolin modifies DNA demethylation and epigenetically promotes Nrf2 expression of the anti-oxidative stress pathway, helping to prevent neoplasm formation in JB6 P+ cells (Vaidya et al., 2020).

In another study, Taxifolin shown strong binding affinity for Kelch-like ECH-associated protein 1 (Keap-1) and HO-1, down-regulating Keap-1 while up-regulating the levels of protective Nrf-2 and linked HO-1 and NQO1 proteins. The study results demonstrated that taxifolin has a protective effect due to its antioxidant effects, which include inhibiting lipid peroxidation, increasing enzymatic and decreasing non-enzymatic anti-oxidative markers, and up-regulating protective HO-1, NQO1, and Nrf-2 expressions, following suppression of Keap-1 mRNA expression (Manigandan et al., 2014).

#### 3.1.1 Taxifolin and colon cancer

Additionally, taxifolin treatment promoted apoptosis and cell cycle arrest in the G2 phase in both colorectal cancer cell lines (HCT116 and HT29) and the HCT116 xenograft model by reducing the expression of the Wnt/β-catenin gene, AKT gene, surviving gene, and protein. This ultimately resulted in a defense regarding colon cancer (Razak et al., 2018).

Taxifolin’s chemo preventive properties were also reported by Manigandan et al. (2014). Taxifolin (4 µg/kg bw, op) triggered antioxidant mediated apoptosis and caused histological changes of cancer cells. Moreover, taxifolin controls cell division and enhances DNA fragmentation. Taxifolin dramatically decreased the incidence of colon cancer in comparison to 5-FU (Tonelli et al., 2018).

#### 3.1.2 Taxifolin and breast cancer

According to both *in vitro* and *in vivo* evaluations, taxifolin is essential for reducing the invasion, migration, and proliferation of cancer cells in aggressive breast carcinoma (Chen et al., 2018). The research' findings indicate that taxifolin significantly and dose-dependently suppresses the migration and proliferation of cancer cells. It also promotes MET (mesenchymal to epithelial transition), inhibits the Epithelial-to-mesenchymal transition (EMT) process, and lowers the expression of mesenchymal markers. Additionally, it lowers β-catenin’s mRNA and protein expressions. Furthermore, as demonstrated in the 4T1 xenograft mouse model, it inhibits the growth of original tumors and stops breast cancer from spreading to the lung (Masciale et al., 2019; Wang et al., 2020).

In Sprague-Dawley rats, taxifolin’s possible chemotherapeutic efficacy against DMBA-induced breast cancer has also been assessed. By significantly restoring the cancer-induced change that promotes tumor growth, the researchers in this study demonstrated how taxifolin alters energy regulation in rats treated with the carcinogen. By blocking the AhR signaling pathway, taxifolin also results in the downregulation of CYP1A1 and CYP1B1 expression (Córdoba et al., 2015). This implied that taxifolin might be employed as a chemotherapeutic drug to suppress DMBA-induced mammary carcinogenesis in a rat model and to combat CYP1A1 and CYP1B1-mediated malignancies.

In numerous research pertaining to tumors, taxifolin has been shown to both directly and indirectly decrease stemness and EMT. In addition to altering a variety of bioactive substances, taxifolin encourages mesenchymal stem cells generated from the human umbilical cord to differentiate into osteoblasts (Li et al., 2019). Additionally, taxifolin has been shown to improve the suppression of the NF-κB signaling pathway linked to osteogenic differentiation. Numerous signaling pathways, including the Janus family tyrosine kinase (JAK)/signal transducer and activator of transcription (STAT)/JAK, Notch, Phosphoinositide 3-kinase (PI3K)/AKT serine/threonine kinase, SHH, and Wnt/β-catenin pathways, are implicated in the control of stemness (Haque and Pattanayak, 2018).

By reducing cluster of differentiation (CD)133-positive cells and downregulating the protein expression of SOX2 and OCT4, taxifolin inhibits stemness. Additionally, taxifolin increased the expression of E-cadherin while suppressing the invasiveness of cancer stem cells and the expression of vimentin and N-cadherin. This demonstrates how taxifolin inhibits EMT from occurring.

#### 3.1.3 Taxifolin and prostate cancer

It is uncommon to find reports of taxifolin combined with other flavonoids. Prostate cancer DU145 cells were used in a recent study to assess the impact of taxifolin and andrographolide (Andro), a diterpenoid lactone that was extracted from the useful plant Andrographis paniculata. Through mitotic phase arrest and intrinsic apoptotic pathway activation, the study demonstrated the impact of Andro in suppressing the proliferation of prostate cancer cells. By increasing mitotic arrest and death through the cleavage of poly (ADP-ribose) polymerase and caspases-7 and -9, the combination of taxifolin with Andro dramatically intensifies the antiproliferative action. In addition to increasing *in-vitro* microtubule polymerization, taxifolin and Andro caused cancer cells to develop twisted and elongated spindles, which ultimately resulted in mitotic arrest. Spindle assembly checkpoint (SAC) component MAD2 was reduced, which enhanced the mitotic block and activated SAC, which resulted in mitotic arrest. Overall, it was proposed that the combination of taxifolin and andrographolide therapy would destabilize microtubule dynamics by activating the SAC (Zhang et al., 2013).

One possible substance that prevents testosterone production is taxifolin. Taxifolin dramatically reduced the amount of androgen produced by Leydig cells in response to pregnenolone, progesterone, LH, 8BR, and basal stimulation. Additionally, taxifolin decreased the activity of the enzymes 17α-hydroxylase/17, 20-lyase and 3β-hydroxysteroid dehydrogenase in both human and rat testes. Based on these results, taxifolin was identified as a possible competitive inhibitor of these two enzymes, which may be useful in the management of prostate cancer (Ge et al., 2018).

AKT serine/threonine kinase 1 (AKT), phosphorylated (p-Ser473) AKT, v-myc avian myelocytomatosis viral oncogene homolog (c-myc), and S-phase kinase associated protein 2 (SKP-2) expression were also significantly downregulated in both cell lines upon treatment with taxifolin. These results indicated that taxifolin could be used to treat osteosarcoma because it inhibits cellular migration and invasion, which is significantly linked to a reduction in SKP-2 overexpression (Haque and Pattanayak, 2018).

### 3.2 Antioxidant activity

The structural variation of flavonoids has a significant impact on their antioxidant potency. It has been demonstrated that the number of hydroxyl groups connected to the aromatic rings substantially boosts antioxidant potential, making it a crucial component of this activity (Zeng et al., 2020). Owing to its phenolic hydroxyl groups, taxifolin, a naturally existing flavonoid, exhibits higher antioxidant efficacy than several conventional flavonoids (Sunil and Xu, 2019).

To effectively counteract free radicals, taxifolin has a 4-oxo functional group and hydroxyl groups at positions 5 and 7 on the A- and C-rings. Its remarkable antioxidant capability is further reinforced by the resonance stability between its two phenolic rings and a conjugated framework. In contrast to other flavonoids with the same hydroxylation pattern, the absence of a double bond between C2 and C3 in the C-ring slightly lowers its antioxidant efficacy (Trouillas et al., 2004).

Taxifolin exploits multiple pathways to produce powerful antioxidant effects. It scavenges ROS such as superoxide, hydroxyl radicals, and peroxy-nitrite. It contributes electrons to stabilize these radicals to halt oxidative damage to proteins, DNA, and lipids (Sa et al., 2018; Arutyunyan et al., 2013). Through the inhibition of both the chain lipid peroxidation and cytochrome c/cardiolipin complex, which led to the release of cytochrome c from mitochondria and the capacity to suppress apoptosis, taxifolin prohibited the production of free radicals (Vladimirov et al., 2009). Topal et al. established that steric freedom and the availability of -OH groups are necessary for antiradical action which was founded on the structure-activity link. The antioxidant activity is increased by a rise in the number of -OH particularly at the aromatic ring’s para location. Besides, a study by Guo et al. found that taxifolin therapy decreases angiotensin II-mediated hypertrophy, ROS production, and protein formation in heart muscle cells (Guo et al., 2015).

Additionally, taxifolin serves as a metal chelator by binding transition metals like Fe^2+^ and Cu^2+^, which activates Fenton reactions that discharge ROS (Li et al., 2017). Moreover, it improves the intrinsic antioxidant mechanisms by upregulating endogenous enzymes such as superoxide dismutase (SOD), catalase, and glutathione peroxidase, and activating the Nrf2 pathway resulting in augmented expression of antioxidant response elements-modulated genes (Algefare, 2022; Jiang et al., 2023; Sun et al., 2014). According to a study by Manigandan et al., Zebrafish embryos treated with taxifolin showed protection against cadmium-mediated toxicity, which is indicated by an elevated development, reduced phenotypic anomalies, diminished heart rate, decreased lipid peroxidation, and higher levels of antioxidant enzymes (Manigandan et al., 2015).

### 3.3 Anti-inflammatory activity

**FIGURE 4:**
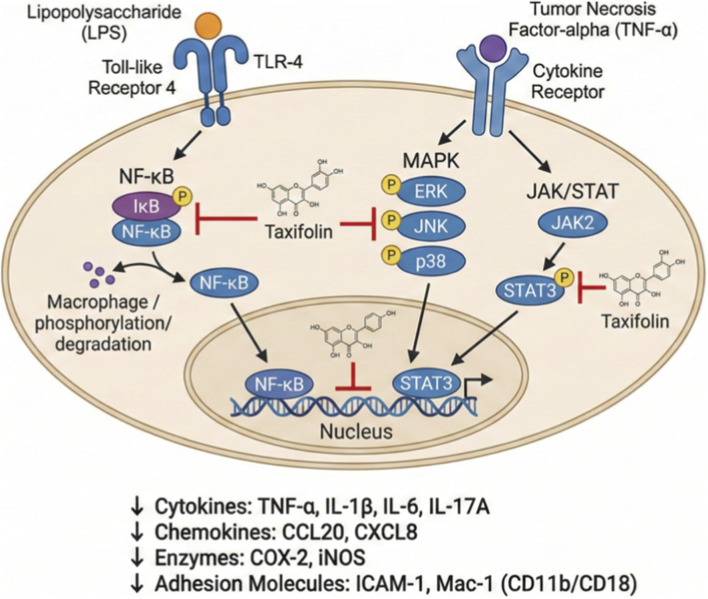
Mechanism of anti-inflammatory activity of taxifolin.

Taxifolin mitigates inflammation through several pathways (Figure 4). It represses the expression of key mediators of systemic inflammation, comprising tumour necrosis factor-alpha (TNF-α), interleukin (IL)-1β, and IL-6 (Sarg et al., 2024; Lei et al., 2020). Using mice and raw 264.7 cells exposed to lipopolysaccharide (LPS) endotoxin, the function of taxifolin in controlling the inflammatory reaction in endotoxemia was examined by Lei et al. research. The findings showed that the gene expression of TNF-α, IL-10, IFN-γ, and toll-like receptor-4 (TLR-4) was considerably reduced by taxifolin administration (Lei et al., 2020). By suppressing Inhibitor of kappa B (IκB)/STAT3 protein phosphorylation in response to TNF-α, IL-17A, and IFN-γ activation in human keratinocytes, taxifolin reduced the expression levels of IL-6, IL-1α/β, chemokine (C-C motif) ligand 20 (CCL20), and chemokine (C-X-C motif) ligand 8 (CXCL8) (Park et al., 2023).

Interestingly, taxifolin suppresses the activation of NF-κB, a transcription element that is crucial for the generation of numerous inflammatory genes, and eventually reduces expression levels of cyclooxygenase-2 (COX-2) and inducible nitric oxide synthase (iNOS), lowering prostaglandin E2 and nitric oxide levels (Wang et al., 2006). Notably, the bone marrow-derived mast cell degranulation, the expression of COX-2, and the synthesis of leukotriene C4 and IL-6 were all reported to be suppressed by taxifolin. Through targeting the Akt/IKK/NF-κB axis, rat basophilic leukaemia and human mast cell stimulation were hindered by taxifolin (Pan et al., 2019).

Taxifolin has been shown to have protective impacts in a mouse model of lipopolysaccharide-mediated bone lysis and to suppress the bone formation of human bone marrow-derived macrophages stimulated by RANKL, receptor activator of NF-κB ligand. Also, it represses RANK ligand-mediated gene expression, comprising the matrix metalloproteinase-9, cathepsin K, nuclear factor of activated T cells 1, tartrate-resistant acid phosphatase, and the production of F-actin rings (Zhang et al., 2019).

Additionally, taxifolin modulates extracellular signal-regulated kinase (ERK), c-Jun N-terminal kinase (JNK), and p38 MAPK pathways, which are stimulated in response to stress and inflammatory stimuli (Zhang et al., 2022a; Olędzka and Czerwińska, 2023). According to some studies, taxifolin lowers systemic and local inflammation by reducing macrophage stimulation and infiltration. The reduced leukocyte infiltration was partly mediated by taxifolin’s inhibition of intercellular adhesion molecule 1 (ICAM-1) and Mac-1 ((CD11b/CD18) expression, two important counter-receptors entailed in leukocyte adherence and transmigration to the endothelium (Wang et al., 2006).

### 3.4 Neuroprotective activity

The main characteristic of Alzheimer’s disease (AD) is the amyloid-*β* (A*β*) peptide’s inherent accumulation and plaque development. The pathophysiology of AD is caused by the accumulation of soluble A*β* oligomeric peptide (A*β*42) (Saini et al., 2019). Taxifolin helps prevent and/or cure cognitive problems linked to A*β*, such as AD and cerebral amyloid angiopathy (CAA) (Tanaka et al., 2019). The deposition of β-amyloid (Aβ) in the endothelium of blood vessels in the brain is a characteristic of CAA, which can result in problems such as cerebral microinfarcts, convexity subarachnoid hemorrhage, and intracerebral hemorrhage (Saito et al., 2021).

The synthesis of amyloid-β in the cerebrum was revealed to be inhibited through blocking of the ApoE-ERK1/2-amyloid-β precursor protein mechanism by taxifolin. Besides, it prohibits the deposition of cells that express TREM2 in the brain. Furthermore, the apoptotic cell death in the brain is suppressed through lowering the production of active caspases, oxidative tissue damage, and glutamate by the introduction of taxifolin (Inoue et al., 2019). Interestingly, the validity of taxifolin had been proved to be a blocking agent for the β-site amyloid precursor protein cleaving enzyme 1 (BACE1), which converts amyloid protein precursor to form Aβ. All the aforementioned mechanisms are involved in the inhibition of the formation of Aβ (Das et al., 2020).

According to Sato et al.'s structure-activity relationship (SAR) investigations, the aggregation of 42-residue amyloid β-protein (Aβ42) can be inhibited by the 3,4-dihydroxyl groups of taxifolin. They also revealed that (+)-taxifolin inhibits A*β*42 aggregation and AD pathogenesis by forming an A*β*42-taxifolin adduct in the presence of sodium periodate (Sato et al., 2013). Recently, taxifolin has been reported to be a therapeutic treatment for AD since it lowers the biochemical and cognitive disorders induced by scopolamine (Chauhan et al., 2025).

Parkinson’s disease, which affects more than 1.6% of people over 65, is the second most prevalent age-related neurodegenerative disorder (Liu et al., 2023a). The neurochemical dysfunction markers, striatal redox stress, and histological features of the brain had been assessed in rat models after the addition of taxifolin to reverse the effect of rotenone. Notably, taxifolin results showed controlling of glutamate metabolism, mitigating the dysfunction in the cholinergic as well as dopaminergic receptors in the striatum, controlling of the dysfunction of mitochondria, and expressing of NF-κBIL-1β and IκKB in parkinsonian rats (Akinmoladun et al., 2022).

Taxifolin showed neuroprotective activity in depressive rates by controlling the Peroxisome proliferator-activated receptor gamma axis, minimizing vacuolization, preserving normal cell shape and size, and reducing brain inflammatory biomarkers (Chauhan et al., 2025). By lowering oxidant status and malondialdehyde concentration and raising total glutathione levels, overall antioxidant status, and superoxide dismutase amount, taxifolin blocked the damage in the sciatic nerve caused by cobalt (Chauhan et al., 2025). The previous effect was also reported in epileptic rat models (Chauhan et al., 2025). Furthermore, neurochemical and histopathological consequences, as well as dementia induced by aluminum chloride, were reversed by treatment of rats with taxifolin (Chauhan et al., 2025). Through the PI3K/AKT signaling pathway, taxifolin suppresses microglial pyroptosis and neuroinflammation following spinal cord injury (SCI). It also enhances functional recovery and encourages axonal regeneration, indicating that taxifolin may be an effective therapy for SCI (Chauhan et al., 2025).

### 3.5 Hepatoprotective activity

Taxifolin has shown promising hepatoprotective effects in various experimental models of liver injury. Its antioxidant and anti-inflammatory properties underpin its ability to counteract oxidative stress and inflammation which are two pivotal mechanisms in the pathogenesis of liver diseases. Taxifolin offers hepatoprotective benefits against several types of liver injuries, particularly those induced by iron overload (Liu et al., 2021). Excessive iron accumulation leads to an increase in ROS, which ultimately contributes to hepatocellular damage (Li et al., 2016). Taxifolin scavenges ferrous ions and free radicals, thereby mitigating oxidative injury and preserving liver function (Salama and Kabel, 2020; Althunibat et al., 2023).

Moreover, taxifolin not only exhibits antioxidative properties but also modulates key signaling pathways, including PI3K/AKT and p38 MAPK, influencing inflammation and promoting hepatocellular regeneration. Several liver biomarkers, including IL-6, IGFBP-2, and MMP-2, are regulated by taxifolin, making them potential indicators for liver diseases (Salama and Kabel, 2020). In addition to oxidative stress modulation, taxifolin alleviates endoplasmic reticulum (ER) stress by reducing the expression of glucose-regulated protein 78 (GRP78) and CHOP, thus mitigating ER stress-induced apoptosis and improving hepatocyte survival (Ezhilarasan and Lakshmi, 2022). Moreover, taxifolin enhances autophagic flux by upregulating autophagy-related proteins such as LC3-II and Beclin-1, promoting the clearance of damaged organelles and lipid droplets, thereby offering further protection against hepatic steatosis and injury (Ding et al., 2022).

Expanding on its protective effects, preclinical studies support the potential use of taxifolin in patients with liver fibrosis. Taxifolin mitigates CCl4-induced liver fibrosis in mice by downregulating collagen I, TyrL, and α-SMA expression while decreasing p-AKT/S6K1, p-mTOR, p66Shc, and ROS levels (Liu et al., 2021). Similarly, in a high-fat diet-induced hepatic steatosis model, taxifolin reversed weight gain and reduced liver steatosis (Inoue et al., 2023; Vu et al., 2024). It inhibited lipid accumulation in hepatocytes, as evidenced by decreased triglyceride levels and histological improvements. The protection is likely mediated by a direct action on hepatocytes to inhibit lipid accumulation, modulation of lipid synthesis pathways, and indirect effects via increased fibroblast growth factor 21 production in the liver (Inoue et al., 2023).

Given the diverse etiologies of liver injury, factors such as drugs, alcohol abuse, malnutrition, viral infections, iron overload, and metabolic disorders contribute to hepatic damage. Carbon tetrachloride (CCl_4_) is commonly used in animal models to induce hepatotoxicity, leading to fatty degeneration, necrosis, and fibrosis of the liver. Fatty liver disease, a common hepatic metabolic disorder, occurs due to excessive lipid accumulation, which is often caused by overproduction of fats, reduced fat oxidation, or impaired lipid transport (Qian and Li, 2025).

Consistently, taxifolin supplementation has been shown to inhibit lipid accumulation in HepG2 hepatocytes through AMPK activation, enhancing fatty acids oxidation and mitochondrial gene expression (Lee et al., 2021). It reduces pro-inflammatory cytokines (e.g., TNF-α, IL-6) and oxidative stress via the Nrf2/HO-1 pathway (Althunibat et al., 2023).

Moreover, taxifolin has demonstrated anti-fibrotic effects in liver injury models, particularly through the inhibition of hepatic stellate cells (HSCs). By modulating the PI3K/AKT/mTOR pathway and transforming growth factor beta 1 (TGF-β1)/Smads signaling, taxifolin reduces collagen deposition and prevents the progression of liver fibrosis and cirrhosis (Liu et al., 2021).

In experimental models, studies have demonstrated that taxifolin effectively counteracts CCl4-induced acute liver injury. Histological and biochemical assessments revealed that taxifolin significantly reduced serum levels of ALT, AST, ALP, and LDH, which are indicative of its hepatoprotective potential. These protective effects were accompanied by improvements in liver function markers and histological architecture, supporting a direct hepatocellular protective role for taxifolin in toxic injury models. Furthermore, pre-treatment with taxifolin (100 mg/kg) significantly reduced liver weight, confirming its protective role against hepatic damage (Yang et al., 2019).

Additionally, taxifolin reduced steatosis area and hepatic triglyceride levels in CCl4-induced liver injury models (Yang et al., 2019). However, further studies employing next-generation sequencing techniques are needed to identify the molecular targets and regulatory pathways through which taxifolin exerts its hepatoprotective effects, especially concerning its impact on steatosis and liver injury.

Beyond its direct hepatic effects, recent studies have highlighted the critical role of the gut-liver axis in the progression of liver diseases. Dysbiosis, or imbalance of the gut microbiota, can lead to increased intestinal permeability, allowing bacterial endotoxins such as LPS to enter the portal circulation and trigger hepatic inflammation (Ming et al., 2024; Albillos et al., 2020). Taxifolin’s hepatoprotective effects may extend to gut-liver axis modulation, as observed in structurally related flavonoids. By hypothetically enhancing intestinal barrier function and reducing LPS translocation, taxifolin could lower systemic inflammation and liver injury. However, direct evidence linking taxifolin to gut microbiota regulation is currently insufficient, and further studies are needed to confirm this mechanism (Ming et al., 2024).

Collectively, preclinical evidence supports a hepatoprotective role for taxifolin across diverse liver injury models, although these findings remain limited to experimental settings and require further validation in chronic and translational models.

### 3.6 Antidiabetic activity

**FIGURE 5:**
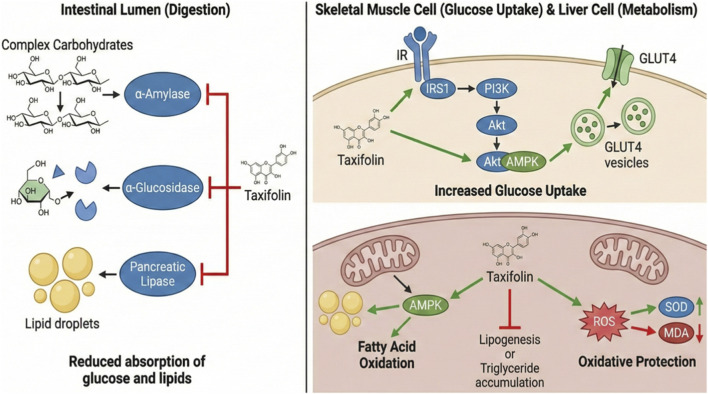
Mechanism of antidiabetic activity of taxifolin.

Taxifolin has demonstrated notable antidiabetic potential in a range of preclinical studies (Figure 5). Several animal models have shown its ability to improve glucose metabolism, enhance insulin sensitivity, and protect against diabetes-related organ damage.

In animal models including KK-Ay/Ta type 2 diabetic mice, taxifolin significantly reduced fasting plasma glucose, insulin, and uric acid levels, accompanied by improvements in Homeostatic model assessment for insulin resistance (HOMA-IR). These effects were attributed to enhanced glucose uptake in skeletal muscle via activation of the PI3K/Akt and AMPK signaling pathways and promotion of GLUT4 translocation (Kondo et al., 2021).

Similarly, in streptozotocin (STZ)-induced diabetic rats, taxifolin treatment lowered blood glucose and creatinine levels, alleviated renal pathological changes, and modulated Caveolin-1/NF-κB-related pathways, suggesting a renoprotective effect (Zhao et al., 2018). Moreover, taxifolin exerted cardioprotective effects by improving cardiac function, reducing oxidative stress, and inhibiting apoptosis in STZ-induced diabetic mice (Sun et al., 2014). In addition, in STZ-nicotinamide diabetic rats, taxifolin decreased fasting blood glucose and ameliorated liver injury by modulating oxidative stress and apoptotic markers (Khadrawy et al., 2024). Notably, taxifolin showed no signs of acute toxicity even at high doses (up to 500 mg/kg), underscoring its safety for further metabolic research (Kondo et al., 2021; Zhao et al., 2018).

The mechanisms underlying taxifolin’s antidiabetic effects are multifactorial. Its strong antioxidant activity, evidenced by reductions in malondialdehyde (MDA) and enhancements in SOD activity, provides robust protection against oxidative stress associated with diabetes (Sun et al., 2014). Taxifolin further improves glucose uptake by activating the PI3K/Akt and AMPK pathways, leading to increased GLUT4 translocation in muscle cells (Lee et al., 2021; Kondo et al., 2021). Its ability to lower insulin resistance is reflected by reductions in HOMA-IR values without affecting food intake (Kondo et al., 2021). Furthermore, taxifolin positively modulates lipid metabolism by lowering serum cholesterol and triglycerides, potentially through leptin signaling regulation (Kondo et al., 2021). Enhancement of insulin signaling components, including increased expression of IRS1 and GLUT4, further underscores its beneficial role in improving insulin sensitivity (Kondo et al., 2021; Yoon et al., 2019).

Despite this encouraging preclinical data, clinical studies evaluating taxifolin’s antidiabetic efficacy remain lacking. Future clinical trials are essential to confirm its therapeutic potential and establish its role in diabetes management.

### 3.7 Cardioprotective activity

Consuming flavonoids on a daily basis reduces the risk of cardiovascular disease, particularly hypertension, and has cardioprotective activity. Flavonoids diminish oxidative stress in endothelial cells or inhibit vascular ion channel activation through boosting the bioavailability of nitric oxide (NO). Six distinct flavonoids, anthocyanins, flavonols, isoflavones, flavanones, and flavones, have been shown by Maaliki et al. to have antihypertensive and cardioprotective properties (Maaliki et al., 2019).

The antihypertensive impact of quercetin mediated by antioxidants was investigated by Duarte et al. (2001). The antihypertensive properties of six distinct flavonoids from three distinct plants were compared by Ahmed et al. (2005). The blood pressure was lowered to 30, 36.5, and 20 mmHg after oral administration of 3.3 mg/kg of 5-hydroxy-3,4′,7-trimethoxyflavone, isoaromadendrin, and taxifolin, respectively. Additionally, the blood pressure was reduced in hypertensive rats through enhancing vasodilation and inhibiting the contraction process, boosting the elevation of anti-inflammatory mechanisms, and weakening COX2-induced pro-inflammation (Jasenovec et al., 2022). Notably, the antihypertensive activity of angiotensin-converting enzyme 2 (ACE2) inhibitors was restored to some extent in hypertensive rats treated with taxifolin (Liskova et al., 2023).

**FIGURE 6:**
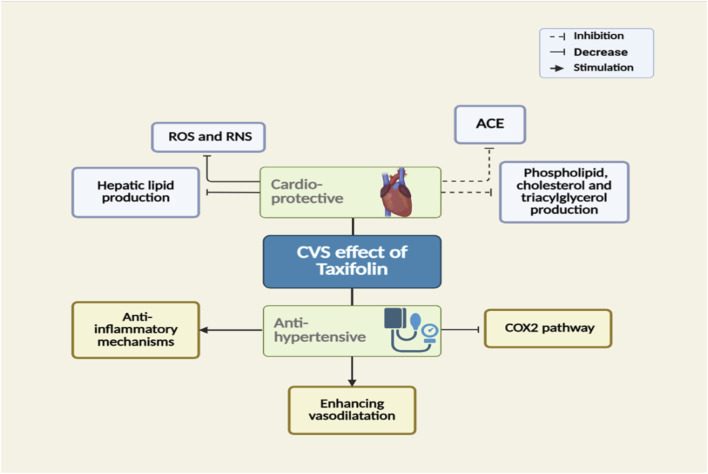
The mechanisms of taxifolin-mediated cardiovascular activity.

Taxifolin may have a function in both preventing and treating cardiovascular disease, according to certain theories (Figure 6). One research study found that taxifolin can decrease hepatic lipid production by lowering and raising apoA-I and apoB secretion, as well as prevent the production of cholesterol in HepG2 cells (Liu et al., 2023a). Additionally, taxifolin strongly suppresses the formation of phospholipids, triacylglycerol, and cholesterol esterification in cells. Furthermore, there is a protective effect of taxifolin against ischemic-reperfusion damage by triggering the PI3K/Akt mechanism (Shu et al., 2019). According to Arutyunyan et al., taxifolin inhibits the angiotensin-converting enzyme (ACE) and the production of ROS/Reactive nitrogen species (RNS) *in vivo*. The findings of the investigation showed that taxifolin administration (100 *μ*g/kg/day) considerably reduced the aortic ACE activity and greatly increased the activity of the enzyme, which in turn triggered the vascular remodeling, causing a lowering of the formation of ROS/RNS. In addition, the researchers proposed that taxifolin had significantly better benefits on cardiovascular disease than quercetin (Arutyunyan et al., 2013).

The cardiac necrosis of di-(2-ethyl hexyl) phthalate (DEHP) was proved to be induced through accumulation of calcium ions in the myocardium, an effect that was opposed by taxifolin (Zheng et al., 2020). The cardiac masturbation of DEHP was revealed to arise through the IL-6/JAK/STAT3 mechanism, which mediates disorder of extracorporeal mitochondria and disruption of glucose metabolism (Cai et al., 2019).

Furthermore, taxifolin treatment was reported to antagonize the cardiac intoxication activity of 5-fluorouracil in rat heart cells. Interestingly, it reduces cardiac MDA and nitric oxide as well as augmenting the antioxidative mechanisms in the myocardium tissues. Besides, the anti-inflammatory response was diminished in the heart through lowering the production of the NO, pro-inflammatory cytokines, and NF-κB after the addition of taxifolin (Abukhalil et al., 2025). Moreover, taxifolin treatment was revealed to mitigate the myocardiac damage induced by clozapine through lowering the cardiac proinflammatory agents such as TNF-α, IL-1β, and NF-B, and levels as well as cardiac enzyme levels such as creatine kinase- MB, Troponin I, and MDA (Cim and Suleyman, 2024). Similarly, taxifolin reverses the acute myocardium damage triggered by isoproterenol through stimulating the Nrf2/HO-1 mechanism, ameliorating the oxidative damage in tissue and key drivers of inflammation and apoptosis (Obeidat et al., 2022). The improvement in heart tissue histology and proper production of cardiac enzymes and cytokines was reported to be achieved by utilization of taxifolin to reverse the detrimental effect of diazinon and Acrylamide (Coşgun et al., 2022; Najeb et al., 2022).

### 3.8 Taxifolin and kidney disorders

ROS generation is inhibited by taxifolin (Ahiskali et al., 2019). According to reports, a key element of taxifolin’s protective impact mechanism is the suppression of proinflammatory cytokines like TNF-α and NF-κB (Akinmoladun et al., 2020). Other than NF-κB, taxifolin has been shown to suppress the overproduction of TNF-α and IL-1β (Ali et al., 2018). Additionally, it has been shown that taxifolin inhibits the invasion of polymorphonuclear leucocytes (Cai et al., 2018). Taxifolin was proposed as a potential treatment for acrylamide-induced oxidative and proinflammatory kidney damage.

In the renal tissues of animals given acrylamide, which had low total glutathione (tGSH) and high MDA levels, the levels of TNF-α and IL-1β were found to be considerably greater than those of the taxifolin and healthy groups. According to Abdelazim et al., acrylamide decreased antioxidant levels in the kidney and other organ tissues while raising IL-1β and TNF-α levels (Masola et al., 2018). According to a different study that backed up our experimental findings, ROS enhanced the synthesis of proinflammatory cytokines (Unver et al., 2019). The results of the experiment from the kidney tissue of the group that received taxifolin further confirmed that proinflammatory cytokines are linked to the balance of oxidants and antioxidants.

It is established that a key element of taxifolin’s protective impact mechanism is its inhibition of the overproduction of proinflammatory cytokines, including NF-κB, IL-1β, and TNF-α (Ahiskali et al., 2019; Akinmoladun et al., 2020). One of the key metrics used to assess renal injury and dysfunction brought on by acrylamide is creatinine and blood urea nitrogen (BUN). However, BUN and creatinine levels that are too high are recognized to be a sign of irreversible renal tubule damage. The antioxidant action of taxifolin may be the reason why the creatinine and BUN levels in the taxifolin group were nearly normal (Li et al., 2017).

Collectively, these findings indicate that the renoprotective effects of taxifolin are primarily mediated through attenuation of oxidative stress and inflammatory cytokine signaling rather than kidney-specific molecular targets.

### 3.9 Cosmetic applications

#### 3.9.1 Anti-aging properties

The skin aging process involves multiple factors that are strongly affected by oxidative stress resulting from an imbalance between reactive oxygen species production and neutralization. Taxifolin demonstrates strong antioxidant properties that make it effective at eliminating dangerous free radicals which help counteract this process (Table 2). The ability of Taxifolin to neutralize damaging molecules serves as a key factor for its investigation in anti-aging skincare products. Taxifolin acts as an antioxidant to protect both skin cells and structural proteins including collagen and elastin from oxidative damage that leads to wrinkles and skin elasticity loss and other aging indicators (Liu et al., 2023a).

**TABLE 2:** Summary of *in vitro* studies evaluating the dermatological and cosmetic-related effects of taxifolin.

Study focus	Cell line/Model	Taxifolin concentration	Key mechanisms	Key findings	References
Anti-aging	Human dermal fibroblasts (NHDF), keratinocytes (NHEK)	1–75 μM	Prevention of ROS generation, glutathione depletion, single-strand breaks, caspase-3 activation; stimulation of Nrf2 translocation and antioxidant protein expression	Effective UVA-protective properties; Taxifolin demonstrated protection across the whole concentration range, while quercetin showed pro-oxidative potential at high concentrations	Rajnochová Svobodová et al. (2017)
Skin Brightening	Murine melanoma B16F10 cells	Not specified	Inhibition of tyrosinase enzymatic activity	Inhibited cellular melanogenesis effectively as arbutin despite increasing tyrosinase protein levels	An et al. (2008)
Hair Growth Promotion	Human follicle dermal papilla cells (HFDPC)	1–50 µg/mL	Antioxidant activity, increased Insulin-like growth factor 1 expression, decreased TGF-β1 expression, inhibition of dihydrotestosterone production	Effectively regulated apoptosis, increased hair growth factor, reduced hair loss biomarker, and showed dose-dependent dihydrotestostero-ne inhibition (less potent than minoxidil). No cytotoxicity observed up to nearly 50 µg/mL	Park et al. (2022)
Psoriasis	HaCaT human keratinocytes	Not specified	Inhibition of mRNA expression of pro-inflammatory cytokines (IL-1α, IL-1-β, IL-6) and chemokines (CXCL8, CCL20) by inhibiting IκB/STAT3 protein phosphorylation	Potential for development as a treatment for psoriasis and skin inflammation by regulating inflammatory cytokine gene expression	Kim et al. (2008)

Besides its antioxidant activity, it is also reported that taxifolin may play a role in the regulation of elastase and collagenase which are responsible for the breakdown of elastin and collagen, the structural proteins that provide skin with its elasticity and firmness. By potentially inhibiting the activity of these enzymes, Taxifolin could help to preserve the structural integrity of the skin over time, offering a mechanism for long-term anti-aging effects that extend beyond its immediate antioxidant action (Drouet et al., 2019). Additionally, Taxifolin was reported to provide protective effects against photoaging because it shields the skin from UV radiation-induced premature aging. Studies demonstrate that Taxifolin reduces the level of melanin by inhibiting melanogenesis thus stopping the development of sun-related wrinkles, fine lines and hyperpigmentation (Rittié and Fisher, 2015).

Moreover, Taxifolin demonstrates potential benefits for strengthening the skin barrier function. *Stizolophus balsamita* extract, which contains Taxifolin as its primary flavonoid component, has been suggested to have the ability to decrease trans-epidermal water loss. Trans-epidermal water loss is the process by which water evaporates from the skin, and a reduction in trans-epidermal water loss indicates an improvement in the skin’s barrier function. By minimizing water loss, Taxifolin can help the skin to retain moisture, leading to improved hydration, increased suppleness, and an overall healthier appearance (Nawrot et al., 2021).

A recent study included 97 Caucasian women with aging skin signs to evaluate how a 3% Taxifolin cream affected their skin parameters. The Taxifolin cream treatment led to a statistically significant enhancement of skin viscoelasticity, the biomechanical property of viscoelasticity measures how skin stretches before returning to its original shape which indicates its firmness and elasticity. The observed increase in viscoelasticity indicates that Taxifolin cream applied topically enhances aging skin mechanical properties which may reduce sagging and wrinkles (Liu et al., 2023a).

Furthermore, the same study observed that both 3% taxifolin cream and 3% *S. balsamita* extract cream containing Taxifolin as the primary flavonoid successfully decreased hyperpigmentation according to the melanin index and reduced skin redness through erythema reduction. Taxifolin demonstrates its effectiveness in treating multiple visible signs of skin aging because it simultaneously enhances skin texture and minimizes unwanted skin discoloration. Taxifolin shows its ability to reduce hyperpigmentation through its inhibition of melanogenesis, while the reduction in erythema likely stems from its anti-inflammatory properties. The penetration rate of the 3% Taxifolin cream surpassed that of the 3% *S. balsamita* extract cream when applied to the skin. The ability of a topical ingredient to permeate the skin barrier is a crucial factor determining its effectiveness. A higher penetration rate ensures that a greater concentration of the active compound reaches the target layers of the skin, where it can exert its beneficial effects. In the context of anti-aging, this enhanced penetration allows more taxifolin to reach the dermis, where collagen and elastin are located, potentially leading to more pronounced anti-aging outcomes (Liu et al., 2023a). The available scientific literature consistently reports a low level of toxicity associated with taxifolin (An et al., 2008; Micek et al., 2021). The positive safety characteristics of Taxifolin make it suitable for cosmetic products that need to be applied topically. The 3% Taxifolin cream demonstrated no irritant effects during patch tests which were conducted on healthy subjects and patients with eczema (Micek et al., 2021). The results demonstrate that taxifolin maintains biosafety properties when used topically on skin regardless of its barrier integrity. Taxifolin demonstrates photostability properties which differ from the phototoxic behavior of quercetin and its related flavonoid structure (Rajnochová Svobodová et al., 2017). Taxifolin demonstrates a crucial benefit for cosmetic ingredients because it indicates that sunscreen products containing this compound will not trigger adverse reactions when exposed to sunlight thus ensuring daytime safety.

#### 3.9.2 Therapeutic effects on skin disorders

##### 3.9.2.1 Eczema and dermatitis

Taxifolin was observed to have anti-inflammatory properties, which are highly relevant to the treatment of inflammatory skin disorders such as eczema (atopic dermatitis) and various other forms of dermatitis (Ahn et al., 2010). The inherent ability of Taxifolin to reduce inflammation is exerted through reducing the expression of chemokines (CCL20 and CXCL8) and pro-inflammatory cytokines (IL-1 α, IL-1-β and IL-6) (Ebrahim et al., 2024). Taxifolin’s capacity to suppress inflammation could therefore help in alleviating these distressing symptoms (Ahn et al., 2010; Ebrahim et al., 2024).

###### 3.9.2.1.1 Psoriasis

Psoriasis is a chronic, immune-mediated skin disease characterized by inflammation and an accelerated rate of skin cell production. Helper T cells (Th) are known to play a critical role in the pathogenesis of this condition (Hu et al., 2021). Taxifolin has shown to attenuate imiquimod induced murine psoriasis-like dermatitis by modulating T helper cell responses through the Notch1 and JAK2/STAT3 signaling pathways (Yuan et al., 2020). T cell differentiation and activation through the Notch1 and JAK2/STAT3 signaling pathways leads to psoriasis-related inflammation (Yuan et al., 2020; Auderset et al., 2012). The inhibition of these pathways by Taxifolin shows potential to decrease the skin-based immune response which contributes to the disease progression.

Additionally, Taxifolin was found to reduce the levels of pro-inflammatory T helper (Th)1 and Th17 cells in both the skin lesions and the skin-draining lymph nodes of the mice. These subtypes of Th cells known to produce cytokines that drive keratinocyte hyperproliferation and inflammation are characteristic features of psoriasis (Yuan et al., 2020). Furthermore, Taxifolin was observed to block the functional activity of transcription factors T-bet, GATA-3, and retinoid-related orphan receptor gamma t (RORγt). The differentiation of Th1, Th2 and Th17 cells depends on these transcription factors for their proper development (Yuan et al., 2020; Radu et al., 2025). Taxifolin demonstrates potential as a psoriasis therapy because it targets pro-inflammatory T cells specifically to treat the fundamental immune dysfunction.

##### 3.9.2.2 Wound healing

Taxifolin has demonstrated a significant role in promoting wound healing through various mechanisms (Liu et al., 2023b; Terekhov et al., 2021). Its inherent antioxidant and anti-inflammatory properties are key contributors to this effect, as these activities help to create an environment conducive to tissue repair (Ding et al., 2023). Studies using Taxifolin-loaded sodium alginate/poly (vinyl alcohol) nanofiber mats in diabetic wound healing models have shown promising results, including the promotion of cell proliferation (indicated by Ki67 expression) and angiogenesis, indicated by CD31 and Vascular endothelial growth factor A (VEGFA) expression. The processes serve as fundamental requirements for proper wound healing and tissue restoration, especially in diabetic ulcers that present healing challenges. Taxifolin exhibits its ability to control wound inflammation through its effects on CD68 expression which serves as a marker for macrophages. The presence of Taxifolin affects the skin flora composition at wound sites by making the microbial ecosystem more diverse while correcting structural abnormalities that help protect against infections and enhance healing. Taxifolin blocks the Toll-like receptor 4/Nuclear factor-kappa B/NOD-, LRR- and pyrin domain-containing protein 3 (TLR4/NF-κB/NLRP3) signaling pathway at the molecular level while simultaneously increasing VEGFA and Platelet-derived growth factor A, growth factors that drive tissue repair through angiogenesis (Wang Y. et al., 2023). Studies indicate Taxifolin-based preparations with liposome complexes demonstrate potential to enhance skin healing and restore hair follicles and sebaceous glands after chemical burn injuries (Liu et al., 2023b).

##### 3.9.2.3 Other skin conditions

Beyond psoriasis, dermatitis, and wound healing, Taxifolin has shown potential in addressing other skin conditions. Laboratory evidence shows Taxifolin inhibits lipase activity while acting as an antioxidant thus indicating potential benefits for acne treatment which involves sebum production and bacterial infection (Liu et al., 2023b). Furthermore, Taxifolin has been considered for its potential in protecting against UV-induced skin carcinogenesis. Studies indicate that it can target key pathways like Epidermal growth factor receptor and Phosphoinositide 3-kinase, which are involved in the development of skin cancer (Micek et al., 2021). Additionally, there is evidence suggesting a protective effect of Taxifolin against cadmium-induced apoptosis in human keratinocytes, which could have implications for the treatment of skin ulcers (Liu et al., 2023b).

#### 3.9.3 Therapeutic effects on hair disorders

Classically, hair loss is caused by a variety of complex mechanisms, including oxidative stress. Oxidative stress is known to cause apoptosis, which stimulates many cell types in the scalp and hair components, including keratinocytes, hair follicle cells, papilla cells, and immune cells (Wang W. et al., 2023).

Taxifolin derived from *Rhododendron mucrotulatum* through enzymatic hydrolysis demonstrated promising hair growth promotion through its ability to control dermal papilla cell apoptosis which exists at hair follicle bases and drives hair development. Taxifolin demonstrates strong antioxidant properties which enable it to control apoptosis. The survival of essential hair follicle cells depends on proper protection against premature death which supports both follicle health and hair growth. The application of Taxifolin elevated insulin-like growth factor 1 concentrations, responsible for hair growth stimulation, while simultaneously decreasing transforming growth factor beta 1 levels, associated with hair loss, in the human dermal papilla cells. This suggests that Taxifolin positively influences key signaling molecules that play a role in the hair growth cycle (Park et al., 2022).

Interestingly, taxifolin treatment also led to a reduction in the inhibition of dihydrotestosterone, a major hormone implicated in androgenetic alopecia. However, this effect was less manifest when compared to that observed with minoxidil (Park et al., 2022). Furthermore, dihydroquercetin, a glycoside derivative of Taxifolin, was also found to produce substantial hair density improvements while simultaneously decreasing hair loss. Ex-vivo studies with hair plucks from androgenetic alopecia patients demonstrated that dihydroquercetin extends the length of hair follicles (Sadgrove et al., 2023).

### 3.10 Antibacterial activity

**FIGURE 7:**
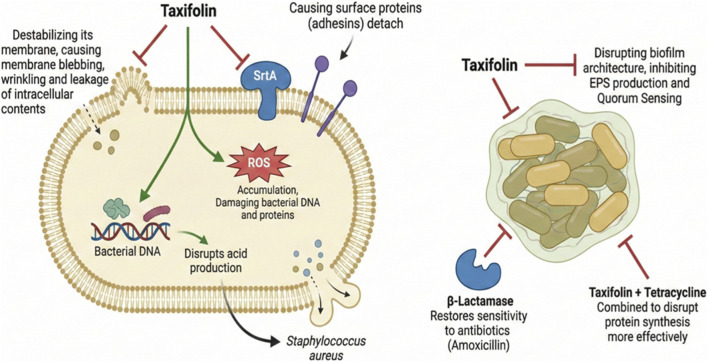
Mechanism of antibacterial activity of taxifolin.

Taxifolin has attracted considerable scientific interest due to its broad-spectrum antibacterial potential (Figure 7) (Abid et al., 2022; Unver, 2024). With rising concerns regarding multi-drug-resistant bacterial strains and biofilm-associated infections, Taxifolin stands out for its multifaceted antibacterial effects, favorable safety profile, and compatibility with standard antimicrobial therapies. As a naturally occurring compound, it aligns well with the growing interest in phytochemicals as therapeutic agents, offering a potential solution to pressing global health challenges (Liu et al., 2023a).

Taxifolin’s antibacterial effects are attributed to several distinct mechanisms, each targeting crucial aspects of bacterial physiology and pathogenicity:

Cell Wall and Membrane Disruption: Taxifolin destabilizes the bacterial membrane structure by increasing membrane permeability, which leads to leakage of essential intracellular contents. Microscopic analyses of treated bacterial cells reveal significant morphological alterations, such as membrane blebbing, wrinkling, and cell lysis, indicative of its lytic action (Unver, 2024; Asmi et al., 2017).

Sortase A (SrtA) Inhibition: In Gram-positive bacteria like *Staphylococcus aureus*, Taxifolin inhibits Sortase A (SrtA), an enzyme pivotal in anchoring surface proteins to the bacterial cell wall. These surface proteins play an essential role in adhesion, immune evasion, and biofilm formation. Inhibiting SrtA thereby impairs bacterial colonization, reduces virulence, and hinders the formation of persistent infections (Wang et al., 2021).

Anti-Biofilm Properties: Biofilms, complex microbial communities encased in a self-produced matrix, protect bacteria from antibiotics and host defenses. Taxifolin disrupts both early-stage biofilm development and mature biofilm architecture by interfering with cell adhesion, extracellular polymeric substance (EPS) production, and quorum sensing pathways (Grabski and Tiratsuyan, 2018; Mu et al., 2021).

Oxidative Stress Induction: Some studies suggest that Taxifolin may enhance ROS generation in bacterial cells, contributing to oxidative damage of proteins, lipids, and nucleic acids (Yang et al., 2023).

Extensive *in vitro* experiments have demonstrated taxifolin’s potent broad-spectrum antibacterial activity against a wide range of bacterial pathogens, including both Gram-positive and Gram-negative strains. It is particularly effective against *S. aureus*, including methicillin-resistant (MRSA) and vancomycin-resistant (VRSA) strains, with minimum inhibitory concentrations (MICs) as low as 0.556 mg/mL, significantly reducing planktonic growth, biofilm formation, and virulence gene expression (Wang et al., 2021). Taxifolin also exhibits notable inhibitory effects against *Escherichia coli*, a Gram-negative bacterium, with MICs around 1.11 mg/mL, likely through mechanisms that compromise bacterial membrane integrity. Furthermore, it effectively targets *Streptococcus mutans*, a key contributor to dental plaque and caries, at an MIC of approximately 1 mg/mL by interfering with acid production and bacterial adhesion. Preliminary studies have also shown its efficacy against *Listeria monocytogenes* and *Bacillus subtilis*, highlighting Taxifolin’s potential application in food preservation and safety (Unver, 2024; Donadio et al., 2021).

One of the most promising aspects of Taxifolin’s antibacterial action is its synergy with conventional antibiotics, which can enhance efficacy, reduce required antibiotic doses, minimize side effects, and delay resistance development.

Tetracycline Combination: When combined with tetracycline, taxifolin significantly enhances antibacterial activity, as demonstrated by fractional inhibitory concentration index (FICI) values that indicate synergy. This co-administration disrupts bacterial protein synthesis more effectively than either agent alone (Yang et al., 2023).

Inhibition of β-Lactamase Activity: In strains of *Pseudomonas aeruginosa* expressing metallo-β-lactamases, Taxifolin restores the activity of β-lactam antibiotics such as amoxicillin, especially when paired with β-lactamase inhibitors like clavulanate. This suggests Taxifolin could function as an adjuvant in antibiotic therapy (Benin et al., 2023).

Potentiation of Multi-Drug Regimens: Preliminary results suggest Taxifolin may also augment the effects of other antibiotic classes, including macrolides and aminoglycosides, although further studies are needed to confirm these findings (Oh and Jeon, 2015).

A critical advantage of taxifolin is its low toxicity to mammalian cells, making it a favorable candidate for therapeutic development. *In vitro* cytotoxicity assays conducted on human epithelial (HEK293) and hepatic (HepG2) cell lines have confirmed its safety at concentrations effective against bacterial pathogens (Rajnochová Svobodová et al., 2017; Wang et al., 2021; Butt et al., 2021). Taxifolin also exhibits antioxidant and anti-inflammatory properties, which may confer additional protective effects to host tissues during bacterial infection. Its selectivity index, the ratio of cytotoxic to antimicrobial concentrations, further supports its potential for clinical use (Park et al., 2023; Liskova et al., 2023).

### 3.11 Antiparasitic activity

Taxifolin has recently gained interest as a potential antiparasitic agent due to the rising global burden of parasitic diseases and resistance to existing therapies. Additionally, its immunomodulatory, antioxidant, and metabolic interference properties make it a promising multi-target antiparasitic candidate (Siheri et al., 2019).

The antiparasitic effects of Taxifolin likely stem from a combination of direct action on parasites and modulation of host cellular responses. Several mechanisms have been proposed:

Redox imbalance modulation: Many parasites rely on delicate redox balances for survival. Taxifolin, through its powerful antioxidant activity, can perturb this balance, creating oxidative stress within the parasite or neutralizing ROS required for parasite proliferation. This disruption may impair parasite replication and increase their susceptibility to immune responses (Jain and Vaidya, 2023).

Enzyme inhibition: Molecular docking and enzyme inhibition studies have demonstrated Taxifolin’s binding affinity to parasite-specific targets such as trypanothione reductase, dihydrofolate reductase, and ornithine decarboxylase. Inhibiting these enzymes disrupts critical biosynthetic and redox pathways, thereby hindering parasite metabolism and survival (Gervazoni et al., 2020; Ogungbe and Setzer, 2016; Gundampati and Jagannadham, 2012).

Membrane disruption and apoptosis: By integrating into lipid membranes, Taxifolin may impair membrane fluidity and permeability, contributing to parasite cell lysis or mitochondrial-induced apoptosis. This is particularly relevant in protozoa, where mitochondrial integrity is crucial for energy metabolism (Abugri et al., 2018; Abugri et al., 2023).

#### 3.11.1 Antiprotozoal activity

Efficacy against Plasmodium spp. (Malaria Parasite) and Trypanosoma spp. (Chagas Disease and African Trypanosomiasis): Taxifolin shows promising antiparasitic activity, inhibiting *Plasmodium falciparum* by disrupting hemozoin formation, mitochondrial function, and inducing oxidative stress, with computational studies highlighting interactions with PfLDH and falcipain-2; limited *in vivo* data suggest partial parasitemia suppression. It also exhibits potential against *Trypanosoma cruzi* and *T. brucei*, with strong *in silico* binding to trypanothione reductase and moderate *in vitro* growth inhibition, possibly enhanced when combined with benznidazole (Siheri et al., 2019; Ganesh et al., 2012).

Impact on Leishmania spp. (Leishmaniasis): Taxifolin exhibits leishmanicidal activity against both promastigote and amastigote stages of *Leishmania*, likely through arginase inhibition, disruption of polyamine synthesis, and enhancement of nitric oxide-mediated macrophage responses. *In vivo* studies in *Leishmania major*-infected mice show reduced lesion size and parasite load, supporting its potential as a therapeutic candidate pending broader validation (Gervazoni et al., 2020; Carter et al., 2021).

### 3.12 Antiviral activity

Taxifolin has emerged as a promising antiviral compound due to its capacity to target multiple aspects of viral pathogenesis. Its ability to regulate key inflammatory and oxidative pathways further contributes to limiting viral proliferation and associated tissue damage. These multifunctional properties support its potential role in the development of novel antiviral therapies (Jiménez-Avalos et al., 2020).

Taxifolin’s antiviral effects are mediated through a combination of direct and indirect mechanisms that collectively inhibit various stages of the viral lifecycle and enhance host antiviral defense:

Inhibition of Viral Replication: Taxifolin can inhibit the function of viral enzymes such as RNA-dependent RNA polymerase (RdRp), DNA polymerase, and viral proteases. These enzymes are critical for the synthesis of viral nucleic acids and the maturation of viral proteins. Inhibition of these enzymes halts replication and assembly of progeny virions, thereby interrupting the infection cycle (Zhu et al., 2022; Selim et al., 2023).

Modulation of Innate and Adaptive Immune Responses: Taxifolin has been shown to influence cytokine signaling pathways such as NF-κB, MAPK, and STAT. By reducing levels of pro-inflammatory cytokines (e.g., IL-1β, IL-6, TNF-α) and enhancing antiviral interferon responses (e.g., IFN-α, IFN-β), Taxifolin helps restore immune balance, curtail hyperinflammation, and facilitate effective clearance of viral pathogens (Zhang et al., 2022b).

#### 3.12.1 Taxifolin against DNA viruses

Activity against Herpes Simplex Viruses (HSV-1 and HSV-2): Experimental studies have shown that Taxifolin inhibits HSV-1, a Herpesviridae family virus, partly by downregulating immediate early viral genes like ICP0 and ICP4, which are vital for initiating replication. It also reduces HSV-induced pro-inflammatory mediators and reactive oxygen species, resulting in less cellular damage and better cell viability. These combined effects suggest Taxifolin may both suppress viral replication and reduce inflammation linked to herpesvirus infections (Kim et al., 2021; Gescher et al., 2011).

Potential against Human Papillomavirus (HPV): Although the direct antiviral effects of Taxifolin on HPV remain under investigation, studies on HPV-positive cervical cancer cells show promising results. Taxifolin suppresses the expression of oncogenic HPV proteins E6 and E7, which degrade tumor suppressors’ p53 and pRb. By restoring their function, Taxifolin promotes cell cycle arrest and apoptosis in infected cells. Combined with its antioxidant and anti-inflammatory properties, this supports its potential in managing HPV-related lesions and cancers (Sarg et al., 2024; Almasoudi et al., 2023; Gomes et al., 2022).

#### 3.12.2 Taxifolin against RNA viruses

Inhibition of Influenza Virus Replication: Influenza viruses are major causes of seasonal respiratory infections and global pandemics. Taxifolin exhibits activity against influenza A strains by inhibiting viral surface glycoproteins like neuraminidase and hemagglutinin, which are vital for viral entry and release. Molecular docking supports Taxifolin’s binding to active sites on these proteins, reducing viral infectivity. Additionally, its anti-inflammatory effects help mitigate the cytokine storm in severe influenza, offering both antiviral and immunomodulatory benefits (Li et al., 2025; Kreiser et al., 2022).

Protective Effects against Hepatitis C Virus (HCV): Hepatitis C virus infection is a leading cause of chronic liver disease and hepatocellular carcinoma globally. In silico and biochemical studies indicate that Taxifolin binds key HCV enzymes like NS3/4A protease and NS5B RNA-dependent RNA polymerase, disrupting viral replication. Additionally, Taxifolin boosts hepatic antioxidant capacity, lowers fibrosis markers, and inhibits pro-inflammatory cytokines in hepatocyte models, potentially improving liver function and mitigating HCV-related pathology (Liu et al., 2023a; Polyak et al., 2013; Patel et al., 2021).

Activity against Coronaviruses (Including SARS-CoV-2): During the COVID-19 pandemic, many natural compounds were screened for SARS-CoV-2 inhibition, with Taxifolin emerging as a promising candidate. Molecular docking studies showed strong binding to the viral main protease (3CLpro), spike glycoprotein, and RNA-dependent RNA polymerase (RdRp). Taxifolin may also block spike protein interaction with the ACE2 receptor, preventing viral entry. Moreover, its ability to suppress excessive immune responses like cytokine storms further supports its therapeutic potential against coronavirus infections (Selim et al., 2023; Bernatova and Liskova, 2021; Gogoi et al., 2021).

## 4 Clinical data

There is minimal clinical research available on taxifolin in humans. A recent Japanese retrospective cohort study (n = 62; 36 participants receiving taxifolin 300 mg/day versus 26 controls) reported a modest but statistically significant weight reduction in the taxifolin group over approximately 6 months (mean −1.6 kg vs. −0.3 kg; P = 0.026), with no observed adverse events (Hattori et al., 2025). Taxifolin use was identified as an independent predictor of weight change, and the study also found a correlation between weight loss and increased HDL cholesterol in taxifolin users (Hattori et al., 2025). However, the study was limited by its single-center design, lack of randomization, small sample size, and absence of dietary or physical activity controls.

Similarly, a small, uncontrolled observational study involving older adults with mild cognitive impairment or dementia (n = 16; expanded to 29 in sensitivity analysis) found that daily intake of taxifolin (300 mg/day) was associated with significantly better preservation of cognitive function during the treatment period compared to the pre-treatment phase (Hattori et al., 2023). Improvements were noted in total Montreal Cognitive Assessment (MoCA) scores and executive function and verbal fluency subscales (*P* ≤ 0.02), although no changes were observed in the ADAS-Cog global score (Hattori et al., 2023). This preservation of cognitive function may be linked to taxifolin’s ability to inhibit Aβ aggregation, production, and glycation, explaining for its neuroprotective effects (Liu et al., 2023a). Like the previous study, this investigation was retrospective, nonrandomized, and based on a small sample, limiting its generalizability.

In contrast, a randomized, double-blind, placebo-controlled crossover trial in 28 healthy young adults assessed the effects of a single dose of taxifolin-enriched food. The study found that taxifolin significantly improved performance in mental calculation tasks and reduced perceived mental fatigue compared to placebo (Liu et al., 2023a). Whole-blood transcriptomic analysis revealed upregulation of innate immunity pathways, particularly those associated with granulocytes, following taxifolin intake (Liu et al., 2023a). No serious adverse events were reported in any of these studies.

A 2024 study by Dr. Ramadan Ali explored the protective properties of taxifolin supplements in thrombo-inflammatory diseases, suggesting potential applications in cardiovascular health and healthy aging (Rysenga et al., 2023). This research complements a notable case report from the same year documenting the successful use of taxifolin in treating early-onset of CAA, as demonstrated in a case report of a 42-year-old patient who underwent neurosurgery. This case report highlights a novel clinical application linked to taxifolin’s inhibition of amyloid aggregation (Choi et al., 2024).

Emerging evidence suggests that taxifolin may be effective in managing metabolic syndrome due to its inhibition of digestive enzymes, including α-glucosidase, α-amylase, and pancreatic lipase (Liu et al., 2023a). Additionally, some researchers have proposed taxifolin as a potential treatment for post-resuscitation therapy following COVID-19 pneumonia, owing to its antioxidant and anti-inflammatory properties (Liu et al., 2023a).

In summary, suggestive evidence points to the potential benefits of taxifolin in body mass regulation, cognitive performance, mental fatigue, and possibly thrombo-inflammatory conditions in humans. However, this understanding is still in its early stages, with limited observational data. Current human studies lack robust evidence to fully support these positive effects. Methodological limitations, such as small sample sizes, short study durations, weak placebo controls, and biased sampling (with findings limited to a single race), hinder the generalizability of the results. While no significant adverse events have been reported in short-term studies, long-term safety data in humans remains insufficient. If taxifolin is to fulfill its potential, larger and well-designed clinical trials across diverse populations are urgently needed.

The current clinical evidence base remains at an early stage because it requires further research to confirm its findings. Most available studies use either retrospective or observational designs which study small groups of people and lack proper randomization and blinding methods while they investigate groups who share similar characteristics. The research methods which investigators used create major bias risks which prevent accurate assessment of study results. The current evidence base does not provide enough data to reach final decisions about clinical effectiveness for any medical condition. Taxifolin does not meet current standards to qualify as a therapeutic treatment supported by scientific evidence. Research needs to prioritize conducting large-scale randomized double-blind placebo-controlled clinical trials which have sufficient power to determine endpoints for assessing the clinical value and safety profile of the product.

## 5 Pharmacokinetics of taxifolin

Taxifolin exhibits a pharmacokinetic profile characterized by low oral bioavailability. In rats, intravenous administration at a dose of 15 mg/kg resulted in extremely high plasma concentrations (C_max ≈3.9 × 10^4^ ng/mL) and an area under the curve (AUC) of approximately 1.48 × 10^4^ ng·h/mL, with a distribution half-life (T_1/2_) of about 2.2 h (Yang et al., 2016). In contrast, oral administration at the same dose yielded peak plasma concentrations of only ∼95 ng/mL, with AUC values ranging from 59 to 153 ng·h/mL, corresponding to a markedly low absolute bioavailability of approximately 0.49% in its unformulated (physical powder) state (Yang et al., 2016). Even with formulation improvements such as polyvinylpyrrolidone (PVP)-based nanodispersion, the bioavailability increased marginally, reaching ∼0.75% (Dash et al., 2024).

As detailed in Table 3, unformulated taxifolin exhibits <1% absolute bioavailability (F ≈ 0.49%), with PVP nanodispersion providing a 1.5-fold improvement (F ≈ 0.75%) and selenized liposomes the largest relative enhancement (4.4-fold, F ≈ 2.16% vs. suspension), yet persistent first-pass metabolism limits translational potential. The various formulation strategies which researchers used have led to some improvements in systemic exposure but most cases show only small increases in bioavailability. Selenized liposomes showed the strongest enhancement among the tested systems but this method still faces challenges because of extensive metabolic transformation. The research results demonstrate that taxifolin maintains poor oral bioavailability which acts as a primary pharmacokinetic barrier that prevents its therapeutic development.

**TABLE 3:** Taxifolin PK parameters in rat models.

Route/Formulation	Dose (mg/kg)	C_max_ (ng/mL)	AUC (ng·h/mL)	T1/2 (h)	Absolute F (%)	Relative F (fold vs. free)	References
IV/Solution	15 (IV)	∼39,000	∼14,800	∼2.2	100	—	Yang et al. (2016)
Oral/Free (powder)	15	∼95	59–153	∼4.8–6.0	∼0.49	1.0 (baseline)	Yang et al. (2016)
Oral/PVP nanodispersion	15	—	∼91	∼5	∼0.75	1.53	Dash et al. (2024)
Oral/Zein–caseinate NPs	15	—	—	—	∼0.52	1.06	Li et al. (2023)
Oral/Selenized liposomes	20	581	∼12,010	13	2.16 (216% rel.)	4.43	Qi et al. (2025)

Absolute F (%) = (AUC_oral/AUC_IV) × 100. Fold = F/free powder (0.49%). All oral F < 3%, confirming bottleneck.

Recent research has indicated nonlinearity in the pharmacokinetics of taxifolin in rats at moderate oral doses (10–50 mg/kg) following a single dose, which may have significant implications for dosing strategies (Lakeev et al., 2023). The effect persisted on day 4 after the administration of multiple cumulative oral doses (with the last dose being 25 mg/kg), suggesting a tendency for accumulation with repeated dosing (Lakeev et al., 2023).

Although taxifolin is rapidly absorbed following oral administration, exhibiting a T_max on the order of minutes, the systemic exposure remains minimal due to rapid presystemic metabolism (Lakeev et al., 2023). The apparent oral elimination half-life (∼4.8–6.0 h) is longer than that observed following intravenous administration, reflecting absorption-limited kinetics. These findings suggest that while taxifolin is absorbed quickly, extensive first-pass metabolism significantly reduces its availability in systemic circulation.

To enhance the pharmacokinetic profile of taxifolin, several formulation strategies have been investigated. Taxifolin is poorly water-soluble and exhibits dissolution-limited absorption, indicating that particle size reduction or carrier-based systems may facilitate improved bioavailability. For instance, nanodispersions using polyvinylpyrrolidone (PVP) have been shown to increase oral bioavailability from approximately 0.5%–0.75% in rats (Yang et al., 2016). Similarly, protein-based nanoparticles composed of zein and caseinate increased bioavailability from ∼0.35% to ∼0.52% (Li et al., 2023).

To improve taxifolin’s bioavailability, its administration route in rats was modified by encapsulating it in selenized liposomes, achieving a 216.65% increase in bioavailability in studies since February 2025 (Qi et al., 2025). This represents a significant advancement over previous formulation strategies and is considered a promising approach to address taxifolin’s poor bioavailability.

Additional formulation approaches reported in the literature include the use of cyclodextrin inclusion complexes, lipid-based systems such as microemulsions and liposomes, and solid dispersions—all of which aim to improve solubility and protect the compound from degradation (Liu et al., 2023a). Among these, oil-in-water and water-in-oil microemulsions appear to improve bioavailability by influencing taxifolin metabolism. However, therapeutic use of taxifolin may introduce complications, as these strategies often require restrictive dosing plans to reduce the area under the curve (AUC) of biotransformed metabolites (Lakeev et al., 2023). While these strategies have generally succeeded in increasing circulating taxifolin levels and prolonging systemic exposure, they have not universally addressed the challenge of its extensive metabolic clearance.

To date, no formal pharmacokinetic studies of taxifolin in humans have been published in peer-reviewed literature. The only available data are derived from rodent models, which limits the direct applicability of these findings to human clinical contexts. Notably, the European Food Safety Authority (EFSA) has evaluated taxifolin derived from *Larix gmelinii* as a novel food ingredient and concluded it to be safe at proposed intake levels for use in food supplements, including for the general adult population and children over 9 years of age (Bresson et al., 2017). This evaluation was based on a specific safety margin and exposure assessment, which suggested that the safety margin is minimal. While this supports its safety as a dietary flavonoid, its therapeutic use would require comprehensive clinical assessment and dedicated pharmacokinetic profiling in human subjects. Theoretically, intravenous or alternative non-oral routes could bypass the limitations associated with gastrointestinal absorption; however, such methods are currently impractical for routine clinical applications.

Following absorption, taxifolin undergoes extensive metabolism. In rodent studies, the predominant metabolites detected in plasma and urine are conjugated forms, including sulfates, glucuronides, and methylated derivatives (Sunil and Xu, 2019). Biologically active metabolites of taxifolin, such as aromadendrin and luteolin, have recently been detected in plasma, where they were previously only found in feces, suggesting more complex metabolic pathways than initially thought (Lakeev et al., 2023). Additionally, the gut microbiota contributes to its biotransformation through ring fission and hydration/dehydration reactions in the colon (Topal et al., 2015). Taxifolin and its metabolites are rapidly distributed to various tissues, although concentrations in the brain and heart remain relatively low (Sunil and Xu, 2019).

Due to this extensive presystemic metabolism, only a minimal amount of unchanged taxifolin is excreted—approximately 2%–3% of the administered dose is recovered in excreta, primarily feces, within 24 h (Li et al., 2023). This highlights the predominant role of metabolic clearance in limiting its systemic availability.

In summary, taxifolin is subject to significant intestinal and hepatic metabolism, resulting in low systemic exposure. While novel formulations, particularly selenized liposomes, have shown promising results in enhancing bioavailability, these pharmacokinetic challenges remain a significant consideration for its broader clinical application. Therefore, further research is essential to optimize delivery systems and explore the pharmacological activity of taxifolin metabolites that could enhance its overall therapeutic effects.

## 6 Discussion

Taxifolin has been widely investigated across multiple biological systems, yet the currently available evidence remains fragmented, disease-specific, and largely confined to preclinical models. When synthesizing findings across studies, a unified pattern emerges: taxifolin exerts its effects mainly through antioxidant, anti-inflammatory, and cell-regulatory pathways, consistent with mechanisms repeatedly described throughout the literature. Unlike previous reviews that primarily summarized its pharmacological actions in a system-by-system manner, the present review organizes these findings around the underlying mechanistic pathways, highlighting the recurrent involvement of the Nrf2/HO-1 axis, NF-κB suppression, and MAPK modulation across inflammatory, metabolic, hepatic, and neurodegenerative models (Liu et al., 2021; Chen et al., 2018). This approach allows the identification of mechanistic overlaps and differences that have been overlooked in prior reviews, clarifying taxifolin’s broad but selective pharmacological profile across disease categories.

Across inflammatory, metabolic, neurodegenerative, cardiovascular, hepatic, and infectious diseases, taxifolin consistently reduces oxidative stress by lowering ROS and enhancing endogenous antioxidant defenses, primarily through activation of the Nrf2/HO-1 axis (Córdoba et al., 2015; Zhang et al., 2013). However, the antioxidant effects are highly variable due to differences in dose, stereochemistry, and extraction purity, and most studies rely on acute injury models, limiting generalizability to chronic human conditions.

Taxifolin also modulates inflammation by suppressing NF-κB, lowering TNF-α, IL-1β, and IL-6, and inhibiting MAPK signaling (Saito et al., 2021; Das et al., 2020; Liu et al., 2023b). These pathways appear repeatedly across endotoxemia, dermatologic inflammation, osteoclastogenesis, and macrophage-related models, yet evidence in autoimmune or chronic inflammatory conditions remains limited. Recent findings also indicate dose-dependent suppression of key inflammatory mediators, including TNF-α, COX-2, VEGF, and iNOS, in LPS-challenged cell models, suggesting that while taxifolin’s anti-inflammatory activity is robust, its magnitude varies according to concentration and model type. Prior literature has primarily focused on acute models, with limited evaluation of taxifolin’s efficacy in chronic disorders, autoimmunity, and multi-organ pathologies, representing a critical gap for future research (Liu et al., 2023b).

In neuroprotection, taxifolin demonstrates anti-amyloid, anti-apoptotic, and mitochondrial-protective effects by inhibiting Aβ aggregation, blocking BACE1, reducing caspase activation, and modulating ApoE-ERK1/2 signaling (Salama and Kabel, 2020; Ezhilarasan and Lakshmi, 2022; Ding et al., 2022). However, these findings are largely restricted to Alzheimer’s disease and chemically induced neurotoxicity, while other neurological conditions (e.g., MS, TBI, chronic neuroinflammation) remain insufficiently explored. Interestingly, emerging evidence also reports improved behavioral performance and favorable neurochemical modulation in Parkinson’s disease models, suggesting that the neuroprotective scope of taxifolin may extend beyond Alzheimer’s-focused research (Lee et al., 2021).

In hepatic and metabolic models, taxifolin protects against steatosis, liver injury, and fibrosis by regulating PI3K/Akt/mTOR and TGF-β1/Smad signaling and enhancing antioxidant enzymes (Liu et al., 2021; Ahmed et al., 2005). Yet nearly all hepatic studies employ short-term injury models, and chronic metabolic inflammation characteristic of metabolic-associated steatotic liver disease and type 2 diabetes remains insufficiently evaluated. Consistent with these metabolic effects, several studies demonstrate that taxifolin improves glucose homeostasis, enhances insulin sensitivity, and reduces diabetic metabolic dysfunction through PI3K/Akt and AMPK activation, GLUT4 translocation, and modulation of lipid metabolism, underscoring a broader role in cardiometabolic regulation (69,95,99,100–102].

Cancer studies show that taxifolin inhibits FAS, modulates Nrf2-related epigenetic pathways, suppresses Wnt/β-catenin signaling, and reduces EMT and stemness features across multiple tumor types (Córdoba et al., 2015; Haque and Pattanayak, 2018; Ge et al., 2018; Trouillas et al., 2004). However, most anticancer findings are based on *in vitro* work using supraphysiological concentrations and lack evaluation in tumor microenvironment or long-term *in vivo* models.

Consistent with its anti-inflammatory and redox-modulatory effects, taxifolin also demonstrates nephroprotective properties, particularly in toxin-induced renal injury models, where it attenuates oxidative stress, restores tGSH levels, suppresses cytokine overproduction, and improves BUN and creatinine (Kim et al., 2008; Drouet et al., 2019; Rittié and Fisher, 2015; Micek et al., 2021; Ahn et al., 2010). These renal findings reinforce a unifying mechanism centered on ROS reduction and cytokine suppression across hepatic, renal, cardiovascular, and metabolic tissues.

Taken together, a distinctive aspect of this review is the systematic mapping of molecular mechanisms across distinct pathological contexts, integrating findings that were previously considered in isolation. This enables more precise targeting of future experimental models and therapeutic strategies.

## 7 Conclusion and future directions

Taxifolin exhibits a broad spectrum of pharmacological activities, including antioxidant, anti-inflammatory, anticancer, neuroprotective, hepatoprotective, antidiabetic, cardioprotective, and nephroprotective effects. These activities are mediated through a limited number of interconnected molecular pathways, most notably activation of the Nrf2/HO-1 antioxidant axis, suppression of NF-κB- and MAPK-driven inflammatory signaling, regulation of apoptosis and mitochondrial function, and modulation of metabolic pathways such as PI3K/Akt and AMPK. Collectively, these mechanisms provide a coherent biological framework underlying taxifolin’s reported effects across diverse disease models. Future research should prioritize mechanistically driven and clinically relevant models to better characterize the therapeutic potential of taxifolin. Evaluating nanoformulations or advanced delivery systems may also help overcome poor oral bioavailability and improve translational potential.

## Glossary

**ACE**: Angiotensin-converting enzyme
**AD**: Alzheimer’s disease
**Akt**: Protein kinase B
**AMPK**: AMP-activated protein kinase
**Aβ**: Amyloid beta
**BACE1**: β-site amyloid precursor protein cleaving enzyme 1
**BUN**: Blood urea nitrogen
**CAA**: Cerebral amyloid angiopathy
**CCL20**: Chemokine (C-C motif) ligand 20
**CD**: Cluster of differentiation
**COX-2**: Cyclooxygenase-2
**CXCL8**: Chemokine (C-X-C motif) ligand 8
**DEHP**: Di-(2-ethylhexyl) phthalate
**EMT**: Epithelial to mesenchymal transition
**ER**: Endoplasmic reticulum
ERK: Extracellular signal-regulated kinase
**FAS**: Fatty acid synthase
**tGSH**: Total glutathione
**HO-1**: Heme oxygenase-1
**HOMA-IR**: Homeostatic model assessment for insulin resistance
**iNOS**: Inducible nitric oxide synthase
**IκB**: Inhibitor of kappa B
**IL**: Interleukin
**JAK**: Janus family tyrosine kinase
**Keap-1**: Kelch-like ECH-associated protein 1
**LPS**: Lipopolysaccharide
**MAPK**: Mitogen-activated protein kinase
**MS**: Mass spectrometry
**MDA**: Malondialdehyde
**mRNA**: Messenger ribonucleic acid
**NF-κB**: Nuclear factor kappa B
**NO**: Nitric oxide
**NQO1**: NAD(P)H quinone oxidoreductase 1
**Nrf2**: Nuclear factor erythroid 2–related factor 2
**PI3K**: Phosphoinositide 3-kinase
**ROS**: Reactive oxygen species
**RNS**: Reactive nitrogen species
**SCI**: Spinal cord injury
**SOD**: Superoxide dismutase
**STAT**: Signal transducer and activator of transcription
**TGF-β1**: Transforming growth factor beta 1
**Th**: T helper
**TNF-α**: Tumor necrosis factor alpha
**UV**: Ultraviolet
**VEGFA**: Vascular endothelial growth factor A

## References

[B1] Abid N. Hamad E. Ibrahim M. Abid H. (2022). Antibacterial and antibiofilm activities of taxifolin against vancomycin-resistant *S. aureus* (VRSA). Baghdad J. Biochem. Appl. Biol. Sci. 3, 262–272. 10.47419/bjbabs.v3i04.126

[B2] Abugri D. A. Witola W. H. Russell A. E. Troy R. M. (2018). *In vitro* activity of the interaction between taxifolin (dihydroquercetin) and pyrimethamine against Toxoplasma gondii. Chem. Biol. & Drug Des. 91, 194–201. 10.1111/cbdd.13070 28696589

[B3] Abugri D. A. Wijerathne S. V. Sharma H. N. Ayariga J. A. Napier A. Robertson B. K. (2023). Quercetin inhibits Toxoplasma gondii tachyzoite proliferation and acts synergically with azithromycin. Parasites & Vectors 16, 261. 10.1186/s13071-023-05849-3 37537675 PMC10401810

[B4] Abukhalil M. H. Al-Alami Z. Alfwuaires M. A. Imran M. R. Aladaileh S. H. Althunibat O. Y. (2025). Taxifolin protects against 5-fluorouracil-induced cardiotoxicity in mice through mitigating oxidative stress, inflammation, and apoptosis: possible involvement of Sirt1/Nrf2/HO-1 signaling. Cardiovasc Toxicol. 25, 455–470. 10.1007/s12012-025-09962-w 39827225

[B5] Ahiskali I. Pinar C. L. Kiki M. Cankaya M. Kunak C. S. Altuner D. J. C. (2019). Effect of taxifolin on methanol-induced oxidative and inflammatory optic nerve damage in rats. Cutan. Ocul. Toxicol. 38, 384–389. 10.1080/15569527.2019.1637348 31242797

[B6] Ahmed B. Al Howiriny T. A. Mossa J. S. Tahir K. E. (2005). Isolation, antihypertensive activity and structure activity relationship of flavonoids from three medicinal plants.

[B7] Ahn J. Y. Choi S. E. Jeong M. S. Park K. H. Moon N. J. Joo S. S. (2010). Effect of taxifolin glycoside on atopic dermatitis‐like skin lesions in NC/Nga mice. Phytotherapy Res. 24, 1071–1077. 10.1002/ptr.3084 20041431

[B8] Akinmoladun A. C. Olaniyan O. O. Famusiwa C. D. Josiah S. S. MTJJoB O. Physiology C. (2020). Ameliorative effect of quercetin, catechin, and taxifolin on rotenone-induced testicular and splenic weight gain and oxidative stress in rats. J. Basic Clin. Physiol. Pharmacol. 31. 10.1515/jbcpp-2018-0230 31940286

[B9] Akinmoladun A. C. Famusiwa C. D. Josiah S. S. Lawal A. O. Olaleye M. T. Akindahunsi A. A. (2022). Dihydroquercetin improves rotenone-induced Parkinsonism by regulating NF-κB-mediated inflammation pathway in rats. J. Biochem. Mol. Toxicol. 36, e23022. 10.1002/jbt.23022 35187747

[B10] Albillos A. De Gottardi A. Rescigno M. (2020). The gut-liver axis in liver disease: pathophysiological basis for therapy. J. Hepatology 72, 558–577. 10.1016/j.jhep.2019.10.003 31622696

[B11] Algefare A. I. (2022). Renoprotective and oxidative stress-modulating effects of taxifolin against cadmium-induced nephrotoxicity in mice. Life 12, 1150. 10.3390/life12081150 36013329 PMC9409698

[B12] Ali B. H. Al‐Salam S. Al Suleimani Y. Al Kalbani J. Al Bahlani S. Ashique M. (2018). Curcumin ameliorates kidney function and oxidative stress in experimental chronic kidney disease. Basic Clin. Pharmacol. Toxicol. 122, 65–73. 10.1111/bcpt.12817 28561324

[B13] Almasoudi H. H. Hakami M. A. Alhazmi A. Y. Makkawi M. Alasmari S. Alghamdi Y. S. (2023). Unveiling the multitargeted repurposing potential of taxifolin (dihydroquercetin) in cervical cancer: an extensive MM\GBSA-based screening, and MD simulation study. Med. Oncol. 40, 218. 10.1007/s12032-023-02094-7 37394519

[B14] Almeida PAd Bhering C. A. Alves M. C. Oliveira MAd Raposo N. R. Ferreira A. O. (2016). Development, optimization and validation of an HPLC-PDA method for quantification of taxifolin in the bark extract of Pinus pinaster. J. Braz. Chem. Soc. 27, 1648–1656. 10.5935/0103-5053.20160044

[B15] Althunibat O. Y. Abukhalil M. H. Jghef M. M. Alfwuaires M. A. Algefare A. I. Alsuwayt B. (2023). Hepatoprotective effect of taxifolin on cyclophosphamide-induced oxidative stress, inflammation, and apoptosis in mice: involvement of Nrf2/HO-1 signaling. Biomol. Biomed. 23, 649–660. 10.17305/bb.2022.8743 36762432 PMC10351093

[B16] An S. M. Kim H. J. Kim J. E. Boo Y. C. (2008). Flavonoids, taxifolin and luteolin attenuate cellular melanogenesis despite increasing tyrosinase protein levels. Phytotherapy Res. 22, 1200–1207. 10.1002/ptr.2435 18729255

[B17] Arutyunyan T. V. Korystova A. F. Kublik L. N. Levitman M. Shaposhnikova V. V. Korystov Y. N. (2013). Effects of taxifolin on the activity of angiotensin-converting enzyme and reactive oxygen and nitrogen species in the aorta of aging rats and rats treated with the nitric oxide synthase inhibitor and dexamethasone. Age (Dordr). 35, 2089–2097. 10.1007/s11357-012-9497-4 23271616 PMC3825014

[B18] Asmi K. S. Lakshmi T. Balusamy S. R. Parameswari R. (2017). Therapeutic aspects of taxifolin-an update.

[B19] Auderset F. Coutaz M. Tacchini-Cottier F. (2012). The role of Notch in the differentiation of CD4+ T helper cells. Notch Regul. Immune Syst. 360, 115–134. 10.1007/82_2012_227 22653552

[B20] Benin B. M. Hillyer T. Crugnale A. S. Fulk A. Thomas C. A. Crowder M. W. (2023). Taxifolin as a metallo-β-lactamase inhibitor in combination with augmentin against verona imipenemase 2 expressing Pseudomonas aeruginosa. Microorganisms 11, 2653. 10.3390/microorganisms11112653 38004664 PMC10673258

[B21] Bernatova I. Liskova S. (2021). Mechanisms modified by (−)-epicatechin and taxifolin relevant for the treatment of hypertension and viral infection: Knowledge from preclinical studies. Antioxidants 10, 467. 10.3390/antiox10030467 33809620 PMC8002320

[B22] Brusselmans K. Vrolix R. Verhoeven G. JVJJoBC S. (2005). Induction of cancer cell apoptosis by flavonoids is associated with their ability to inhibit fatty acid synthase activity. J. Biol. Chem. 280, 5636–5645. 10.1074/jbc.M408177200 15533929

[B23] Bresson J. L. Burlingame B. Dean T. Fairweather-Tait S. Heinonen M. Hirsch-Ernst K. I. (2017). Statement on the safety of taxifolin-rich extract from Dahurian Larch (Larix gmelinii). EFSA J. 15, e05059-e. 32625351 10.2903/j.efsa.2017.5059 PMC7010057

[B24] Butt S. S. Khan K. Badshah Y. Rafiq M. Shabbir M. (2021). Evaluation of pro-apoptotic potential of taxifolin against liver cancer. PeerJ 9, e11276. 10.7717/peerj.11276 34113483 PMC8162243

[B25] Cai C. Liu C. Zhao L. Liu H. Li W. Guan H. (2018). Effects of taxifolin on osteoclastogenesis *in vitro* and *in vivo* . Front. Pharmacol. 9, 1286. 10.3389/fphar.2018.01286 30483128 PMC6240596

[B26] Cai J. Shi G. Zhang Y. Zheng Y. Yang J. Liu Q. (2019). Taxifolin ameliorates DEHP-induced cardiomyocyte hypertrophy via attenuating mitochondrial dysfunction and glycometabolism disorder in chicken. Environ. Pollut. 255, 113155. 10.1016/j.envpol.2019.113155 31539850

[B27] Carter N. S. Stamper B. D. Elbarbry F. Nguyen V. Lopez S. Kawasaki Y. (2021). Natural products that target the arginase in Leishmania parasites hold therapeutic promise. Microorganisms 9, 267. 10.3390/microorganisms9020267 33525448 PMC7911663

[B28] Cerezo A. B. Álvarez-Fernández M. A. Hornedo-Ortega R. Troncoso A. M. García-Parrilla M. C. (2014). Phenolic composition of vinegars over an accelerated aging process using different wood species (Acacia, Cherry, Chestnut, and Oak): effect of wood toasting. J. Agric. Food Chem. 62, 4369–4376. 10.1021/jf500654d 24779921

[B29] Chauhan H. Nakum B. Chaube U. Saxena B. (2025). Unravelling the neuroprotective effects of taxifolin against scopolamine-induced dementia in male Sprague Dawley rats: a comprehensive preclinical investigation. Brain Disord. 17, 100203. 10.1016/j.dscb.2025.100203

[B30] Chemical book (2022). Synthesis and application of taxifolin.

[B31] Chen X. Gu N. Xue C. Li B.-R. J. (2018). Plant flavonoid taxifolin inhibits the growth, migration and invasion of human osteosarcoma cells. Mol. Med. Rep. 17, 3239–3245. 10.3892/mmr.2017.8271 29257319

[B32] Choi M. C. Y. Law T. H. P. Chen S. Cheung W. S. K. Yim C. Ng O. K. S. (2024). Case Report: taxifolin for neurosurgery-associated early-onset cerebral amyloid angiopathy. Front. Neurol. 15, 1360705. 10.3389/fneur.2024.1360705 38566852 PMC10985332

[B33] Cim E. F. A. Suleyman H. (2024). Effect of taxifolin on clozapine-induced experimental oxidative and inflammatory heart damage in rats. Annales Médico-psychologiques, revue psychiatrique. Elsevier, 823–829.

[B34] Córdoba A. Satué M. Gómez‐Florit M. Hierro‐Oliva M. Petzold C. Lyngstadaas S. P. (2015). Flavonoid‐modified surfaces: multifunctional bioactive biomaterials with osteopromotive, anti‐inflammatory, and anti‐fibrotic potential. Adv. Healthc. Mater. 4, 540–549. 10.1002/adhm.201400587 25335455

[B35] Coşgun M. S. Çoşkun R. Celik A. I. (2022). The preventive effect of taxifolin on acrylamide-induced heart damage in rats. Rev. Nutr. 35, e210079. 10.1590/1678-9865202235e210079

[B36] Das S. Majumder T. Sarkar A. Mukherjee P. Basu S. (2020). Flavonoids as BACE1 inhibitors: QSAR modelling, screening and *in vitro* evaluation. Int. J. Biol. Macromol. 165, 1323–1330. 10.1016/j.ijbiomac.2020.09.232 33010267

[B37] Das A. Baidya R. Chakraborty T. Samanta A. K. Roy S. (2021). Pharmacological basis and new insights of taxifolin: a comprehensive review. Biomed. & Pharmacother. 142, 112004. 10.1016/j.biopha.2021.112004 34388527

[B38] Dash J. R. Pattnaik G. Samal H. B. Pradhan G. Baral C. P. K. Behera B. (2024). Novel approaches for the enhancement of bioavailability of drugs: an updated review. Curr. Drug Discov. Technol. 21. 10.2174/0115701638311058240806100555 39129281

[B39] De Marino S. Festa C. Zollo F. Nini A. Antenucci L. Raimo G. (2014). Antioxidant activity and chemical components as potential anticancer agents in the olive leaf (Olea europaea L. cv Leccino.) decoction. Anti-Cancer Agents Med. Chemistry-Anti-Cancer Agents 14, 1376–1385. 10.2174/1871520614666140804153936 25102361

[B40] Ding Q. Chen K. Liu X. Ding C. Zhao Y. Sun S. (2022). Modification of taxifolin particles with an enteric coating material promotes repair of acute liver injury in mice through modulation of inflammation and autophagy signaling pathway. Biomed. & Pharmacother. 152, 113242. 10.1016/j.biopha.2022.113242 35691160

[B41] Ding Q. Ding C. Liu X. Zheng Y. Zhao Y. Zhang S. (2023). Preparation of nanocomposite membranes loaded with taxifolin liposome and its mechanism of wound healing in diabetic mice. Int. J. Biol. Macromol. 241, 124537. 10.1016/j.ijbiomac.2023.124537 37086765

[B42] Donadio G. Mensitieri F. Santoro V. Parisi V. Bellone M. L. De Tommasi N. (2021). Interactions with microbial proteins driving the antibacterial activity of flavonoids. Pharmaceutics 13, 660. 10.3390/pharmaceutics13050660 34062983 PMC8147964

[B43] Drouet S. Leclerc E. A. Garros L. Tungmunnithum D. Kabra A. Abbasi B. H. (2019). A green ultrasound-assisted extraction optimization of the natural antioxidant and anti-aging flavonolignans from milk thistle Silybum marianum (L.) gaertn. fruits for cosmetic applications. Antioxidants 8, 304. 10.3390/antiox8080304 31416140 PMC6721202

[B44] Duarte J. Pérez-Palencia R. Vargas F. Ocete M. A. Pérez-Vizcaino F. Zarzuelo A. (2001). Antihypertensive effects of the flavonoid quercetin in spontaneously hypertensive rats. Br. J. Pharmacol. 133, 117–124. 10.1038/sj.bjp.0704064 11325801 PMC1572775

[B45] Ebrahimi A. Mehrabi M. Miraghaee S. S. Mohammadi P. Kafash F. F. Delfani M. (2024). Flavonoid compounds and their synergistic effects: promising approaches for the prevention and treatment of psoriasis with emphasis on keratinocytes–A systematic and mechanistic review. Int. Immunopharmacol. 138, 112561. 10.1016/j.intimp.2024.112561 38941673

[B46] Ezhilarasan D. Lakshmi T. (2022). A molecular insight into the role of antioxidants in nonalcoholic fatty liver diseases. Oxidative Med. Cell. Longev. 2022, 9233650. 10.1155/2022/9233650 35602098 PMC9117022

[B47] Ganesh D. Fuehrer H.-P. Starzengrüber P. Swoboda P. Khan W. A. Reismann J. A. (2012). Antiplasmodial activity of flavonol quercetin and its analogues in Plasmodium falciparum: evidence from clinical isolates in Bangladesh and standardized parasite clones. Parasitol. Res. 110, 2289–2295. 10.1007/s00436-011-2763-z 22215188

[B48] Ge F. Tian E. Wang L. Li X. Zhu Q. Wang Y. (2018). Taxifolin suppresses rat and human testicular androgen biosynthetic enzymes. Fitoterapia 125, 258–265. 10.1016/j.fitote.2018.01.017 29402482

[B49] Gervazoni L. F. Barcellos G. B. Ferreira-Paes T. Almeida-Amaral E. E. (2020). Use of natural products in leishmaniasis chemotherapy: an overview. Front. Chemistry 8, 579891. 10.3389/fchem.2020.579891 33330368 PMC7732490

[B50] Gescher K. Kühn J. Hafezi W. Louis A. Derksen A. Deters A. (2011). Inhibition of viral adsorption and penetration by an aqueous extract from Rhododendron ferrugineum L. as antiviral principle against herpes simplex virus type-1. Fitoterapia 82, 408–413. 10.1016/j.fitote.2010.11.022 21129454

[B51] Gogoi N. Chowdhury P. Goswami A. K. Das A. Chetia D. Gogoi B. (2021). Computational guided identification of a citrus flavonoid as potential inhibitor of SARS-CoV-2 main protease. Mol. Diversity 25, 1745–1759. 10.1007/s11030-020-10150-x 33236176 PMC7685905

[B52] Gomes D. Yaduvanshi S. Silvestre S. Duarte A. P. Santos A. O. Soares C. P. (2022). Taxifolin and lucidin as potential e6 protein inhibitors: P53 function re-establishment and apoptosis induction in cervical cancer cells. Cancers 14, 2834. 10.3390/cancers14122834 35740499 PMC9221127

[B53] Grabski H. Tiratsuyan S. (2018). Mechanistic insights of the attenuation of quorum-sensing-dependent virulence factors of Pseudomonas aeruginosa: molecular modeling of the interaction of taxifolin with transcriptional regulator LasR. bioRxiv, 500157.

[B54] Gundampati R. K. Jagannadham M. V. (2012). Molecular docking based inhibition of trypanothione reductase activity by taxifolin novel target for antileishmanial activity. J. Appl. Pharm. Sci. 2, 133–136.

[B55] Guo H. Zhang X. Cui Y. Zhou H. Xu D. Shan T. (2015). Taxifolin protects against cardiac hypertrophy and fibrosis during biomechanical stress of pressure overload. Toxicol. Appl. Pharmacol. 287, 168–177. 10.1016/j.taap.2015.06.002 26051872

[B56] Hammerbacher A. Kandasamy D. Ullah C. Schmidt A. Wright L. P. Gershenzon J. (2019). Flavanone-3-Hydroxylase plays an important role in the biosynthesis of spruce phenolic defenses against bark beetles and their fungal associates. Front. Plant Sci. 10, 208. 10.3389/fpls.2019.00208 30858861 PMC6397876

[B57] Haque M. W. Siddique M. U. M. Bose P. Pattanayak SPJPM (2018). Taxifolin possesses anti-cancer activity on the 7, 12-Dimethylbenz (a) anthracene-Induced breast cancer in the sprague dawley rats by remodeling nuclear factor Erythroid 2-Kelch-like ECH-associated protein 1-heme oxygenase 1 and anti-oxidant pathways. 14.

[B58] Haque M. W. Pattanayak S. P. (2018). Taxifolin inhibits 7, 12-dimethylbenz (a) anthracene-induced breast carcinogenesis by regulating AhR/CYP1A1 signaling pathway. 13:S749. 29491628 10.4103/pm.pm_315_17 PMC5822495

[B59] Hattori Y. Saito S. Nakaoku Y. Ogata S. Hattori M. Nakatsuji M. (2023). Taxifolin for cognitive preservation in patients with mild cognitive impairment or mild dementia. J. Alzheimer’s Dis. 93, 743–754. 10.3233/JAD-221293 37092223 PMC10200220

[B60] Hattori Y. Nakaoku Y. Ogata S. Saito S. Nishimura K. Ihara M. (2025). Taxifolin as a therapeutic potential for weight loss: a retrospective longitudinal study. Nutrients 17, 706. 10.3390/nu17040706 40005033 PMC11858263

[B61] Hu P. Wang M. Gao H. Zheng A. Li J. Mu D. (2021). The role of helper T cells in psoriasis. Front. Immunology 12, 788940. 10.3389/fimmu.2021.788940 34975883 PMC8714744

[B62] Inoue T. Saito S. Tanaka M. Yamakage H. Kusakabe T. Shimatsu A. (2019). Pleiotropic neuroprotective effects of taxifolin in cerebral amyloid angiopathy. Proc. Natl. Acad. Sci. U. S. A. 116, 10031–10038. 10.1073/pnas.1901659116 31036637 PMC6525485

[B63] Inoue T. Fu B. Nishio M. Tanaka M. Kato H. Tanaka M. (2023). Novel therapeutic potentials of taxifolin for obesity-induced hepatic steatosis, fibrogenesis, and tumorigenesis. Nutrients 15, 350. 10.3390/nu15020350 36678220 PMC9865844

[B64] Jain S. Vaidya A. (2023). Comprehensive review on pharmacological effects and mechanism of actions of taxifolin: a bioactive flavonoid. Pharmacol. Research-Modern Chin. Med. 7, 100240. 10.1016/j.prmcm.2023.100240

[B65] Jasenovec T. Radosinska D. Kollarova M. Balis P. Zorad S. Vrbjar N. (2022). Effects of taxifolin in spontaneously hypertensive rats with a focus on erythrocyte quality. Life (Basel) 12, 2045. 10.3390/life12122045 36556410 PMC9788412

[B66] Jiang H. Yu J. Yan Z. Lin Z. Lin M. Mao Y. (2023). Pharmacological activation of the Nrf2 pathway by Taxifolin remodels articular cartilage microenvironment for the therapy of Osteoarthritis. Int. Immunopharmacol. 122, 110587. 10.1016/j.intimp.2023.110587 37399606

[B67] Jiménez-Avalos G. M. Vargas-Ruiz A. P. Delgado-Pease N. E. Olivos-Ramirez G. E. Sheen-Cortavarría P. Fernandez M. (2020). High-throughput virtual screening of 4487 flavonoids: new insights on the structural inhibition of SARS-CoV-2 main protease. arXiv Preprint arXiv:200813264.

[B68] Khadrawy S. M. Altoom N. G. Alotaibi A. G. Othman S. I. (2024). Hepatoprotective potential of taxifolin in type 2 diabetic rats: modulation of oxidative stress and Bcl2/Bax/Caspase-3 signaling pathway. Mol. Biol. Rep. 51, 897. 10.1007/s11033-024-09805-x 39115553

[B69] Kiehlmann E. Li E. P. (1995). Isomerization of dihydroquercetin. J. Natural Products 58, 450–455. 10.1021/np50117a018

[B70] Kim Y. J. Choi S. E. Lee M. W. Lee C. S. (2008). Taxifolin glycoside inhibits dendritic cell responses stimulated by lipopolysaccharide and lipoteichoic acid. J. Pharm. Pharmacol. 60, 1465–1472. 10.1211/jpp/60.11.0007 18957167

[B71] Kim B. Kim Y. S. Hwang Y.-H. Yang H. J. Li W. Kwon E.-B. (2021). Quercus acuta thunb.(Fagaceae) and its component, isoquercitrin, inhibit HSV-1 replication by suppressing virus-induced ROS production and NF-κB activation. Antioxidants 10, 1638. 10.3390/antiox10101638 34679772 PMC8533069

[B72] Kim J. Han S. H. Kim N. K. Tran G. H. Shim J. Chin J. H. (2024). Antioxidant activities and silymarin content of Silybum marianum using different extraction methods. J. Appl. Biol. Chem. 67, 397–406. 10.3839/jabc.2024.055

[B73] Kondo S. Adachi S. I. Yoshizawa F. Yagasaki K. (2021). Antidiabetic effect of taxifolin in cultured L6 myotubes and type 2 diabetic model KK-ay/Ta mice with hyperglycemia and hyperuricemia. Curr. Issues Mol. Biol. 43, 1293–1306. 10.3390/cimb43030092 34698101 PMC8929065

[B74] Kreiser T. Zaguri D. Sachdeva S. Zamostiano R. Mograbi J. Segal D. (2022). Inhibition of respiratory RNA viruses by a composition of ionophoric polyphenols with metal ions. Pharmaceuticals 15, 377. 10.3390/ph15030377 35337174 PMC8955458

[B75] Kuang H. Tang Z. Zhang C. Wang Z. Li W. Yang C. (2017). Taxifolin activates the Nrf2 anti-oxidative stress pathway in mouse skin epidermal JB6 P+ cells through epigenetic modifications. Int. J. Mol. Sci. 18, 1546. 10.3390/ijms18071546 28714938 PMC5536034

[B76] Kumar S. Baldi A. Sharma D. K. (2021). *In vitro* antioxidant assay guided *ex vivo* investigation of cytotoxic effect of phytosomes assimilating taxifolin rich fraction of Cedrus deodara bark extract on human breast cancer cell lines (MCF7). J. Drug Deliv. Sci. Technol. 63, 102486. 10.1016/j.jddst.2021.102486

[B77] Lakeev A. P. Yanovskaya E. A. Yanovsky V. A. Frelikh G. A. Andropov M. O. (2023). Novel aspects of taxifolin pharmacokinetics: dose proportionality, cumulative effect, metabolism, microemulsion dosage forms. J. Pharm. Biomed. Analysis 236, 115744. 10.1016/j.jpba.2023.115744 37797493

[B78] Lee S. B. Cha K. H. Selenge D. Solongo A. Nho C. W. (2007). The chemopreventive effect of taxifolin is exerted through ARE-dependent gene regulation. Biol. Pharm. Bull. 30, 1074–1079. 10.1248/bpb.30.1074 17541156

[B79] Lee H. Jeong W.-T. So Y.-S. Lim H.-B. Lee J. (2021). Taxifolin and sorghum ethanol extract protect against hepatic insulin resistance via the miR-195/IRS1/PI3K/AKT and AMPK signalling pathways. Antioxidants 10, 1331. 10.3390/antiox10091331 34572963 PMC8465682

[B80] Lei L. Chai Y. Lin H. Chen C. Zhao M. Xiong W. (2020). Dihydroquercetin activates AMPK/Nrf2/HO-1 signaling in macrophages and attenuates inflammation in LPS-induced endotoxemic mice. Front. Pharmacology 11, 662. 10.3389/fphar.2020.00662 32508636 PMC7248193

[B81] Li S. W. Liu C. M. Guo J. Marcondes A. M. Deeg J. Li X. (2016). Iron overload induced by ferric ammonium citrate triggers reactive oxygen species-mediated apoptosis via both extrinsic and intrinsic pathways in human hepatic cells. Hum. & Experimental Toxicology 35, 598–607. 10.1177/0960327115597312 26224043

[B82] Li X. Xie H. Jiang Q. Wei G. Lin L. Li C. (2017). The mechanism of (+) taxifolin’s protective antioxidant effect for• OH-treated bone marrow-derived mesenchymal. Stem Cells 22, 1–11. 10.1186/s11658-017-0066-9 PMC5745628 29299033

[B83] Li J. Hu L. Zhou T. Gong X. Jiang R. Li H. (2019). Taxifolin inhibits breast cancer cells proliferation, migration and invasion by promoting mesenchymal to epithelial transition via β-catenin signaling. Life Sci. 232, 116617. 10.1016/j.lfs.2019.116617 31260685

[B84] Li Y. Su H. Wang W. Yin Z. Li Je Yuan E. (2023). Fabrication of taxifolin loaded zein-caseinate nanoparticles and its bioavailability in rat. Food Sci. Hum. Wellness 12, 2306–2313. 10.1016/j.fshw.2023.03.034

[B85] Li H. Shen B. Bi Y. Sun Y. Zhang S. Xue K. (2025). Miquelianin inhibits IAV infection via the MAPK signaling pathway both *in vitro* and *in vivo* . Front. Immunol. 16, 1532336. 10.3389/fimmu.2025.1532336 40165966 PMC11955610

[B86] Liskova S. Cacanyiova S. Cebova M. Berenyiova A. Kluknavsky M. Micurova A. (2023). Taxifolin reduces blood pressure via improvement of vascular function and mitigating the vascular inflammatory response in spontaneously hypertensive rats. Int. J. Mol. Sci. 24, 12616. 10.3390/ijms241612616 37628795 PMC10454553

[B87] Liu W. Feng Y. Yu S. Fan Z. Li X. Li J. (2021). The flavonoid biosynthesis network in plants. Int. J. Mol. Sci. 22, 12824. 10.3390/ijms222312824 34884627 PMC8657439

[B88] Liu Y. Shi X. Tian Y. Zhai S. Liu Y. Xiong Z. (2023a). An insight into novel therapeutic potentials of taxifolin. Front. Pharmacol. 14, 1173855. 10.3389/fphar.2023.1173855 37261284 PMC10227600

[B89] Liu Z. Qiu D. Yang T. Su J. Liu C. Su X. (2023b). Research progress of dihydroquercetin in the treatment of skin diseases. Molecules 28, 6989. 10.3390/molecules28196989 37836832 PMC10574795

[B90] Luo H. Jiang B.-H. King S. M. Chen YCJN (2008). Inhibition of cell growth and VEGF expression in ovarian cancer cells by flavonoids. Nutr. Cancer, 60:800–809. 10.1080/01635580802100851 19005980

[B91] Maaliki D. Shaito A. A. Pintus G. El-Yazbi A. Eid A. H. (2019). Flavonoids in hypertension: a brief review of the underlying mechanisms. Curr. Opin. Pharmacol. 45, 57–65. 10.1016/j.coph.2019.04.014 31102958

[B92] Manigandan K. Jayaraj R. L. Elangovan N. J. B. Nutrition P. (2014). Taxifolin ameliorates 1, 2-dimethylhydrazine induced cell proliferation and redox avulsions in mice colon carcinogenesis. 4:499–509. 10.1016/j.bionut.2014.08.009

[B93] Manigandan K. Jayaraj R. L. Jagatheesh K. Elangovan N. (2015). Taxifolin mitigates oxidative DNA damage *in vitro* and protects zebrafish (Danio rerio) embryos against cadmium toxicity. Environ. Toxicol. Pharmacol. 39, 1252–1261. 10.1016/j.etap.2015.04.021 26002187

[B94] Masciale V. Grisendi G. Banchelli F. D’Amico R. Maiorana A. Sighinolfi P. (2019). Correlating tumor-infiltrating lymphocytes and lung cancer stem cells: a cross-sectional study. Ann. Transl. Med. 7, 619. 10.21037/atm.2019.11.27 31930020 PMC6944548

[B95] Masola B. Oguntibeju O. O. Oyenihi ABJB (2018). Pharmacotherapy. Centella asiatica ameliorates diabetes-induced stress in rat tissues via influences on antioxidants and inflammatory cytokines. 101:447–457. 10.1016/j.biopha.2018.02.115 29501767

[B96] Micek I. Nawrot J. Seraszek-Jaros A. Jenerowicz D. Schroeder G. Spiżewski T. (2021). Taxifolin as a promising ingredient of cosmetics for adult skin. Antioxidants 10, 1625. 10.3390/antiox10101625 34679758 PMC8533573

[B97] Ming Z. Ruishi X. Linyi X. Yonggang Y. Haoming L. Xintian L. (2024). The gut-liver axis in fatty liver disease: role played by natural products. Front. Pharmacology 15, 1365294. 10.3389/fphar.2024.1365294 38686320 PMC11056694

[B98] Moura F. C. S. dos Santos Machado C. L. Paula F. R. Couto A. G. Ricci M. Cechinel-Filho V. (2021). Taxifolin stability: *in silico* prediction and *in vitro* degradation with HPLC-UV/UPLC–ESI-MS monitoring. J. Pharm. Analysis 11, 232–240. 10.1016/j.jpha.2020.06.008 PMC8116214 34012699

[B99] Mu Y. Zeng H. Chen W. (2021). Quercetin inhibits biofilm formation by decreasing the production of EPS and altering the composition of EPS in Staphylococcus epidermidis. Front. Microbiol. 12, 631058. 10.3389/fmicb.2021.631058 33763049 PMC7982815

[B100] Najeb S. M. Jaccob A. A. Al-Moziel M. S. G. Abdulhameed H. M. (2022). Cardioprotective and antioxidant effects of taxifolin and vitamin C against diazinone-induced myocardial injury in rats. Environ. Anal. Health Toxicol. 37, e2022002-0. 10.5620/eaht.2022002 35108778 PMC9058105

[B101] Nawrot J. Budzianowski J. Nowak G. Micek I. Budzianowska A. Gornowicz-Porowska J. (2021). Biologically active compounds in Stizolophus balsamita inflorescences: isolation, phytochemical characterization and effects on the skin biophysical parameters. Int. Journal Molecular Sciences 22, 4428. 10.3390/ijms22094428 33922647 PMC8122880

[B102] Nifant’ev E. Koroteev M. Kaziev G. Uminskii A. Grachev A. Men’shov V. (2006). On the problem of identification of the dihydroquercetin flavonoid. Russ. Journal General Chemistry 76, 161–163. 10.1134/s1070363206010324

[B103] Obeidat H. M. Althunibat O. Y. Alfwuaires M. A. Aladaileh S. H. Algefare A. I. Almuqati A. F. (2022). Cardioprotective effect of taxifolin against isoproterenol-induced cardiac injury through decreasing oxidative stress, inflammation, and cell death, and activating Nrf2/HO-1 in mice. Biomolecules 12, 1546. 10.3390/biom12111546 36358896 PMC9687704

[B104] Ogungbe I. V. Setzer W. N. (2016). The potential of secondary metabolites from plants as drugs or leads against protozoan neglected diseases—Part III: in-silico molecular docking investigations. Molecules 21, 1389. 10.3390/molecules21101389 27775577 PMC6274513

[B105] Oh E. Jeon B. (2015). Synergistic anti-Campylobacter jejuni activity of fluoroquinolone and macrolide antibiotics with phenolic compounds. Front. Microbiology 6, 1129. 10.3389/fmicb.2015.01129 26528273 PMC4602130

[B106] Olędzka A. J. Czerwińska M. E. (2023). Role of plant-derived compounds in the molecular pathways related to inflammation. Int. J. Mol. Sci. 24, 4666. 10.3390/ijms24054666 36902097 PMC10003729

[B107] Pan S. Zhao X. Ji N. Shao C. Fu B. Zhang Z. (2019). Inhibitory effect of taxifolin on mast cell activation and mast cell-mediated allergic inflammatory response. Int. Immunopharmacol. 71, 205–214. 10.1016/j.intimp.2019.03.038 30925321

[B108] Park S. M. He Y. C. Gong C. Gao W. Bae Y. S. Si C. (2022). Effects of taxifolin from enzymatic hydrolysis of Rhododendron mucrotulatum on hair growth promotion. Front. Bioeng. Biotechnol. 10, 995238. 10.3389/fbioe.2022.995238 36159701 PMC9492874

[B109] Park J. E. Kwon H. J. Lee H. J. Hwang H. S. (2023). Anti-inflammatory effect of taxifolin in TNF-α/IL-17A/IFN-γ induced HaCaT human keratinocytes. Appl. Biol. Chem. 66, 8. 10.1186/s13765-023-00769-3

[B110] Patel A. Patel R. Memon M. Desai S. Meshram D. (2021). INSILICO docking and molecular dynamic simulation studies OF NS3 protease from hepatitis C virus. Eur. J. Biomed. 8, 413–421.

[B111] Polyak S. J. Ferenci P. Pawlotsky J. M. (2013). Hepatoprotective and antiviral functions of silymarin components in hepatitis C virus infection. Hepatology 57, 1262–1271. 10.1002/hep.26179 23213025 PMC3594650

[B112] Pozharitskaya O. N. Karlina M. V. Shikov A. N. Kosman V. M. Makarova M. N. Makarov V. G. (2009). Determination and pharmacokinetic study of taxifolin in rabbit plasma by high-performance liquid chromatography. Phytomedicine 16, 244–251. 10.1016/j.phymed.2008.10.002 19110406

[B113] Qi C. Xing H. Ding N. Feng W. Wu Y. Zhang X. (2025). Nanometerizing taxifolin into selenized liposomes to ameliorate its hypoglycemic effect by optimizing drug release and bioavailability. Int. J. Nanomedicine 20, 2225–2240. 10.2147/IJN.S510378 40007903 PMC11853828

[B114] Qian Z. Li J. (2025). Novel insights into liver injury: mechanisms, pathophysiology, and therapeutic strategies. Front. Med. 12, 1542598. 10.3389/fmed.2025.1542598 40041457 PMC11876417

[B115] Rajnochová Svobodová A. Ryšavá A. Psotová M. Kosina P. Zálešák B. Ulrichová J. (2017). The phototoxic potential of the flavonoids, taxifolin and quercetin. Photochem. Photobiol. 93, 1240–1247. 10.1111/php.12755 28303596

[B116] Radu A. Tit D. M. Endres L. M. Radu A.-F. Vesa C. M. Bungau S. G. (2025). Naturally derived bioactive compounds as precision modulators of immune and inflammatory mechanisms in psoriatic conditions. Inflammopharmacology 33, 527–549. 10.1007/s10787-024-01602-z 39576422 PMC11842495

[B117] Razak S. Afsar T. Ullah A. Almajwal A. Alkholief M. Alshamsan A. (2018). Taxifolin, a natural flavonoid interacts with cell cycle regulators causes cell cycle arrest and causes tumor regression by activating Wnt/β-catenin signaling pathway. BMC Cancer 18, 1–18. 10.1186/s12885-018-4959-4 30367624 PMC6204009

[B118] Rittié L. Fisher G. J. (2015). Natural and sun-induced aging of human skin. Cold Spring Harbor Perspectives Medicine 5, a015370. 10.1101/cshperspect.a015370 25561721 PMC4292080

[B119] Rohdewald P. (2002). A review of the French maritime pine bark extract (Pycnogenol), a herbal medication with a diverse clinical pharmacology. Int. Journal Clinical Pharmacology Therapeutics 40, 158–168. 10.5414/cpp40158 11996210

[B120] Rysenga C. E. May-Zhang L. Zahavi M. Knight J. S. Ali R. A. (2023). Taxifolin inhibits NETosis through activation of Nrf2 and provides protective effects in models of lupus and antiphospholipid syndrome. Rheumatology. 63, 2006–2015. 10.1093/rheumatology/kead547 37815837

[B121] Sakuma S. Kishiwaki Y. Matsumura M. Sawada H. Hashimoto R. Gotoh K. (2018). Taxifolin potently diminishes levels of reactive oxygen species in living cells possibly by scavenging peroxyl radicals. Am. J. Pharmacol. Toxicol. 13, 1–6. 10.3844/ajptsp.2018.1.6

[B122] Sadgrove N. Batra S. Barreto D. Rapaport J. (2023). An updated etiology of hair loss and the new cosmeceutical paradigm in therapy: clearing ‘the big eight strikes. Cosmetics 10, 106. 10.3390/cosmetics10040106

[B123] Saini R. K. Shuaib S. Goyal D. Goyal B. (2019). Insights into the inhibitory mechanism of a resveratrol and clioquinol hybrid against Aβ(42) aggregation and protofibril destabilization: a molecular dynamics simulation study. J. Biomol. Struct. Dyn. 37, 3183–3197. 10.1080/07391102.2018.1511475 30582723

[B124] Saito S. Tanaka M. Satoh-Asahara N. Carare R. O. Ihara M. (2021). Taxifolin: a potential therapeutic agent for cerebral amyloid angiopathy. Front. Pharmacol. 12, 643357. 10.3389/fphar.2021.643357 33643053 PMC7907591

[B125] Salama S. A. Kabel A. M. (2020). Taxifolin ameliorates iron overload-induced hepatocellular injury: modulating PI3K/AKT and p38 MAPK signaling, inflammatory response, and hepatocellular regeneration. Chemico-biological Interactions 330, 109230. 10.1016/j.cbi.2020.109230 32828744

[B126] Sarg N. H. Hersi F. H. Zaher D. M. Hamouda A. O. Ibrahim S. I. El-Seedi H. R. (2024). Unveiling the therapeutic potential of Taxifolin in Cancer: from molecular mechanisms to immune modulation and synergistic combinations. Phytomedicine 133, 155934. 10.1016/j.phymed.2024.155934 39128306

[B127] Sato M. Murakami K. Uno M. Ikubo H. Nakagawa Y. Katayama S. (2013). Structure-activity relationship for (+)-taxifolin isolated from silymarin as an inhibitor of amyloid β aggregation. Biosci. Biotechnol. Biochem. 77, 1100–1103. 10.1271/bbb.120925 23649236

[B128] Selim S. Albqmi M. Alanazi A. Alruwaili Y. Al-Sanea M. M. Alnusaire T. S. (2023). Antiviral activities of olive oil apigenin and taxifolin against SARS-CoV-2 RNA-dependent RNA polymerase (RdRP): *in silico*, pharmacokinetic, ADMET, and in-vitro approaches. Cogent Food & Agric. 9, 2236828. 10.1080/23311932.2023.2236828

[B129] Shu Z. Yang Y. Yang L. Jiang H. Yu X. Wang Y. (2019). Cardioprotective effects of dihydroquercetin against ischemia reperfusion injury by inhibiting oxidative stress and endoplasmic reticulum stress-induced apoptosis via the PI3K/Akt pathway. Food Funct. 10, 203–215. 10.1039/c8fo01256c 30525169

[B130] Siheri W. Ebiloma G. U. Igoli J. O. Gray A. I. Biddau M. Akrachalanont P. (2019). Isolation of a novel flavanonol and an alkylresorcinol with highly potent anti-trypanosomal activity from Libyan propolis. Molecules 24, 1041. 10.3390/molecules24061041 30884752 PMC6471328

[B131] Slimestad R. Fossen T. Vågen I. M. (2007). Onions: a source of unique dietary flavonoids. J. Agricultural Food Chemistry 55, 10067–10080. 10.1021/jf0712503 17997520

[B132] Stenger Moura F. C. Pinna N. Vivani R. Nunes G. E. Schoubben A. Bellé Bresolin T. M. (2021). Exploring taxifolin polymorphs: insights on hydrate and anhydrous forms. Pharmaceutics 13, 1328. 10.3390/pharmaceutics13091328 34575404 PMC8469002

[B133] Subramanian S. Ramachandran S. (2022). Isolation, simultaneous quantification of taxifolin and taxifolin-3-O-rhamnoside and validation by RPHPLC. Pharmacogn. Res. 14, 30–34. 10.5530/pres.14.1.6

[B134] Sun X. Chen R.-c. Yang Z.-h. Sun G.-b. Wang M. Ma X.-j. (2014). Taxifolin prevents diabetic cardiomyopathy *in vivo* and *in vitro* by inhibition of oxidative stress and cell apoptosis. Food Chem. Toxicol. 63, 221–232. 10.1016/j.fct.2013.11.013 24269735

[B135] Sunil C. Xu B. (2019). An insight into the health-promoting effects of taxifolin (dihydroquercetin). Phytochemistry 166, 112066. 10.1016/j.phytochem.2019.112066 31325613

[B136] Tanaka M. Saito S. Inoue T. Satoh-Asahara N. Ihara M. (2019). Novel therapeutic potentials of taxifolin for amyloid-β-associated neurodegenerative diseases and other diseases: recent advances and future perspectives. Int. J. Mol. Sci. 20. 10.3390/ijms20092139 31052203 PMC6539020

[B137] Terekhov R. P. Selivanova I. A. Tyukavkina N. A. Ilyasov I. R. Zhevlakova A. K. Dzuban A. V. (2020). Assembling the puzzle of taxifolin polymorphism. Molecules 25, 5437. 10.3390/molecules25225437 33233608 PMC7699767

[B138] Terekhov R. Selivanova I. Anurova M. Zhevlakova A. Nikitin I. Cong Z. (2021). Comparative study of wound-healing activity of dihydroquercetin pseudopolymorphic modifications. Bull. Exp. Biol. Med. 170, 444–447. 10.1007/s10517-021-05083-w 33713223 PMC7955205

[B139] Tonelli C. Chio I. I. C. Tuveson DAJA (2018). Signaling r. Transcriptional regulation by Nrf2. Antioxid. Redox Signal. 29, 1727–1745. 10.1089/ars.2017.7342 28899199 PMC6208165

[B140] Topal F. Nar M. Gocer H. Kalin P. Kocyigit U. M. Gülçin İ. (2015). Antioxidant activity of taxifolin: an activity–structure relationship. J. Enzyme Inhibition Med. Chem. 31, 674–683. 10.3109/14756366.2015.1057723 26147349

[B141] Trouillas P. Fagnère C. Lazzaroni R. Calliste C. Marfak A. Duroux J.-L. (2004). A theoretical study of the conformational behavior and electronic structure of taxifolin correlated with the free radical-scavenging activity. Food Chem. 88, 571–582. 10.1016/j.foodchem.2004.02.009

[B142] Unver T. (2024). The inhibitory effects of taxifolin, namely dihydroquercetin as a pharmaceutical agent on the growth of bacterial and fungal species.

[B143] Unver E. Tosun M. Olmez H. Kuzucu M. Cimen F. K. ZJMoi S. (2019). The effect of taxifolin on cisplatin‐induced pulmonary damage in rats: a biochemical and histopathological evaluation. Mediat. Inflamm. 2019, 3740867. 10.1155/2019/3740867 30992689 PMC6434269

[B144] Usman A. Thoss V. Nur-e-Alam M. (2016). Isolation of taxifolin from Trichilia emetica whole seeds. Amer Sci. Res. J. Eng. Technol. Sci. 21, 77–82.

[B145] Vaidya A. Jain S. Sahu S. Jain P. K. Pathak K. Pathak D. (2020). Anticancer agents based on vulnerable components in a signalling pathway. Mini Rev. Med. Chem. 20, 886–907. 10.2174/1389557520666200212105417 32048968

[B146] Vinayagamurthy K. Kumar D. P. Yalavarthi K. Ramudu K. N. Ravichandran J. Stephen N. M. (2024). Biological significance of polyphenols as functional molecules in biomaterial preparations. Sci. Eng. Polyphenols Fundam. Industrial Scale Appl., 548–583.

[B147] Vladimirov Y. A. Proskurnina E. Demin E. Matveeva N. Lubitskiy O. Novikov A. (2009). Dihydroquercetin (taxifolin) and other flavonoids as inhibitors of free radical formation at key stages of apoptosis. Biochem. Mosc. 74, 301–307. 10.1134/s0006297909030092 19364325

[B148] Vu T. T. Pham T. K. Ngo T. Y. Pham T. B. Luu T. T. P. (2024). Taxifolin attenuates hepatic fibrosis and injury in high-fat diet and streptozotocin-induced type 2 diabetes mice. VNU J. Sci. Nat. Sci. Technol. 40.

[B149] Wallace S. N. Carrier D. J. Clausen E. C. (2005). Batch solvent extraction of flavanolignans from milk thistle (Silybum marianum L. Gaertner). Phytochemical Analysis An Int. J. Plant Chem. Biochem. Tech. 16, 7–16. 10.1002/pca.803 15688950

[B150] Wang Y.-H. Wang W.-Y. Chang C.-C. Liou K.-T. Sung Y.-J. Liao J.-F. (2006). Taxifolin ameliorates cerebral ischemia-reperfusion injury in rats through its anti-oxidative effect and modulation of NF-kappa B activation. J. Biomedical Science 13, 127–141. 10.1007/s11373-005-9031-0 16283433

[B151] Wang R. Zhu X. Wang Q. Li X. Wang E. Zhao Q. (2020). The anti-tumor effect of taxifolin on lung cancer via suppressing stemness and epithelial-mesenchymal transition *in vitro* and oncogenesis in nude mice. Ann. Transl. Med. 8, 590. 10.21037/atm-20-3329 32566617 PMC7290558

[B152] Wang L. Wang G. Qu H. Wang K. Jing S. Guan S. (2021). Taxifolin, an inhibitor of Sortase A, interferes with the adhesion of Methicillin-resistant Staphylococcal aureus. Front. Microbiol. 12, 686864. 10.3389/fmicb.2021.686864 34295320 PMC8290497

[B153] Wang Y. Ding C. Zhao Y. Zhang J. Ding Q. Zhang S. (2023a). Sodium alginate/poly(vinyl alcohol)/taxifolin nanofiber mat promoting diabetic wound healing by modulating the inflammatory response, angiogenesis, and skin flora. Int. J. Biol. Macromol. 252, 126530. 10.1016/j.ijbiomac.2023.126530 37634780

[B154] Wang W. Wang H. Long Y. Li Z. Li J. (2023b). Controlling hair loss by regulating apoptosis in hair follicles: a comprehensive overview. Biomolecules 14, 20. 10.3390/biom14010020 38254620 PMC10813359

[B155] Wei M. Zhao R. Peng X. Feng C. Gu H. Yang L. (2020). Ultrasound-assisted extraction of taxifolin, diosmin, and quercetin from abies nephrolepis (trautv.) maxim: kinetic and thermodynamic characteristics. Molecules 25, 1401. 10.3390/molecules25061401 32204461 PMC7144359

[B156] Weidmann A. E. (2012). Dihydroquercetin: more than just an impurity? Eur. J. Pharmacol. 684, 19–26. 10.1016/j.ejphar.2012.03.035 22513183

[B157] Yang C.-J. Wang Z.-B. Mi Y.-Y. Gao M.-J. Lv J.-N. Meng Y.-H. (2016). UHPLC-MS/MS determination, pharmacokinetic, and bioavailability study of taxifolin in rat plasma after oral administration of its nanodispersion. Molecules 21, 494. 10.3390/molecules21040494 27089318 PMC6273324

[B158] Yang C.-L. Lin Y.-S. Liu K.-F. Peng W.-H. Hsu C.-M. (2019). Hepatoprotective mechanisms of taxifolin on carbon tetrachloride-induced acute liver injury in mice. Nutrients 11, 2655. 10.3390/nu11112655 31689986 PMC6893565

[B159] Yang D. Zhu R. Xu H.-X. Zhang Q.-F. (2023). Antibacterial mechanism of taxifolin and its application in milk preservation. Food Biosci. 53, 102811. 10.1016/j.fbio.2023.102811

[B160] Yoon K. D. Lee J.-Y. Kim T. Y. Kang H. Ha K.-S. Ham T.-H. (2019). *In vitro* and *in vivo* anti-hyperglycemic activities of taxifolin and its derivatives isolated from pigmented rice (Oryzae sativa L. cv. Superhongmi). J. Agricultural Food Chemistry 68, 742–750. 10.1021/acs.jafc.9b04962 31880937

[B161] Yuan X. Li N. Zhang M. Lu C. Du Z. Zhu W. (2020). Taxifolin attenuates IMQ-induced murine psoriasis-like dermatitis by regulating T helper cell responses via Notch1 and JAK2/STAT3 signal pathways. Biomed. & Pharmacother. 123, 109747. 10.1016/j.biopha.2019.109747 31881484

[B162] Zeng Y. Song J. Zhang M. Wang H. Zhang Y. Suo H. (2020). Comparison of *in vitro* and *in vivo* antioxidant activities of six flavonoids with similar structures. Antioxidants 9, 732. 10.3390/antiox9080732 32796543 PMC7465758

[B163] Zhang Z. R. Al Zaharna M. Wong M. M.-K. Chiu S.-K. Cheung H.-YJPO (2013). Taxifolin enhances andrographolide-induced mitotic arrest and apoptosis in human prostate cancer cells via spindle assembly checkpoint activation. PLoS One 8, e54577. 10.1371/journal.pone.0054577 23382917 PMC3557238

[B164] Zhang H.-Q. Wang Y.-J. Yang G.-T. Gao Q.-L. Tang M.-X. (2019). Taxifolin inhibits receptor activator of NF-κB ligand-induced osteoclastogenesis of human bone marrow-derived macrophages *in vitro* and prevents lipopolysaccharide-induced bone loss *in vivo* . Pharmacology 103, 101–109. 10.1159/000495254 30522105

[B165] Zhang X. Lian X. Li H. Zhao W. Li X. Zhou F. (2022a). Taxifolin attenuates inflammation via suppressing MAPK signal pathway *in vitro* and *in silico* analysis. Chin. Herbal Medicines 14, 554–562. 10.1016/j.chmed.2021.03.002 36405054 PMC9669345

[B166] Zhang X. Shan L.-P. Zhao Q. Liu L. OuYang X. Hu Y. (2022b). Taxifolin inhibits WSSV infection and transmission by increasing the innate immune response in Litopenaeus vannamei. Viruses 14, 2731. 10.3390/v14122731 36560735 PMC9787842

[B167] Zhao Y. Huang W. Wang J. Chen Y. Huang W. Zhu Y. (2018). Taxifolin attenuates diabetic nephropathy in streptozotocin-induced diabetic rats. Am. J. Transl. Res. 10, 1205–1210. 29736213 PMC5934579

[B168] Zheng Y. Shi G. Cai J. Yang J. Zhang Y. Gong Y. (2020). Di-(2-ethyl hexyl) phthalate induces necroptosis in chicken cardiomyocytes by triggering calcium overload. J. Hazard Mater 387, 121696. 10.1016/j.jhazmat.2019.121696 31889598

[B169] Zhou S. Shao Y. Fu J. Xiang L. Zheng Y. Li W. (2018). Characterization and quantification of taxifolin related flavonoids in Larix olgensis Henry Var. koreana Nakai extract analysis and its antioxidant activity assay. Int. J. Pharmacol. 14, 534–545. 10.3923/ijp.2018.534.545

[B170] Zhu Y. Scholle F. Kisthardt S. C. Xie D.-Y. (2022). Flavonols and dihydroflavonols inhibit the main protease activity of SARS-CoV-2 and the replication of human coronavirus 229E. Virology 571, 21–33. 10.1016/j.virol.2022.04.005 35439707 PMC9002334

